# Model Substrate/Inactivation Reactions for MoaA and Ribonucleotide Reductases: Loss of Bromo, Chloro, or Tosylate Groups from C2 of 1,5-Dideoxyhomoribofuranoses upon Generation of an α-Oxy Radical at C3 †

**DOI:** 10.3390/molecules25112539

**Published:** 2020-05-29

**Authors:** Stanislaw F. Wnuk, Mukesh M. Mudgal, Ireneusz Nowak, Morris J. Robins

**Affiliations:** 1 Department of Chemistry and Biochemistry, Florida International University, Miami, FL 33199, USA; mmudg001@fiu.edu; 2 Department of Chemistry and Biochemistry, Brigham Young University, Provo, UT 84602-5700, USA; i_nowak@hotmail.com

**Keywords:** mechanism-based enzyme inhibition, ribonucleotide reductases, biomimetic modeling, carbohydrates, radical chemistry, oxyl radicals, biosynthesis of molybdopterin

## Abstract

We report studies on radical-initiated fragmentations of model 1,5-dideoxyhomoribofuranose derivatives with bromo, chloro, and tosyloxy substituents on C2. The effects of stereochemical inversion at C2 were probed with the corresponding arabino epimers. In all cases, the elimination of bromide, chloride, and tosylate anions occurred when the 3-hydroxyl group was unprotected. The isolation of deuterium-labeled furanone products established heterolytic cleavage followed by the transfer of deuterium from labeled tributylstannane. In contrast, 3-*O*-methyl derivatives underwent the elimination of bromine or chlorine radicals to give the 2,3-alkene with no incorporation of label in the methyl vinyl ether. More drastic fragmentation occurred with both of the 3-*O*-methyl-2-tosyloxy epimers to give an aromatized furan derivative with no deuterium label. Contrasting results observed with the present anhydroalditol models relative to our prior studies with analogously substituted nucleoside models have demonstrated that insights from biomimetic chemical reactions can provide illumination of mechanistic pathways employed by ribonucleotide reductases (RNRs) and the MoaA enzyme involved in the biosynthesis of molybdopterin.

## 1. Introduction

Ribonucleotide reductases (RNRs) effect conversion of nucleoside 5′-(di- or tri)phosphates into their 2′-deoxy counterparts and thereby provide the only de novo access to the monomeric precursors for DNA replication and repair [1]. Depletion of these crucial deoxynucleotide pools by inhibition of RNRs presents an inviting approach for rational drug design [2,3]. Mechanism-based inactivation of RNRs has been observed with 2′-chloro [4] and 2′-azido [5,6,7,8] analogues of pyrimidine nucleoside 5′**-**(di- or tri)phosphates, and the potent inactivator 2′-deoxy-2′,2′-difluorocytidine (gemcitabine) is a primary drug for the treatment of pancreatic and non-small cell lung cancers [2,3,9,10].

**Figure 1:**
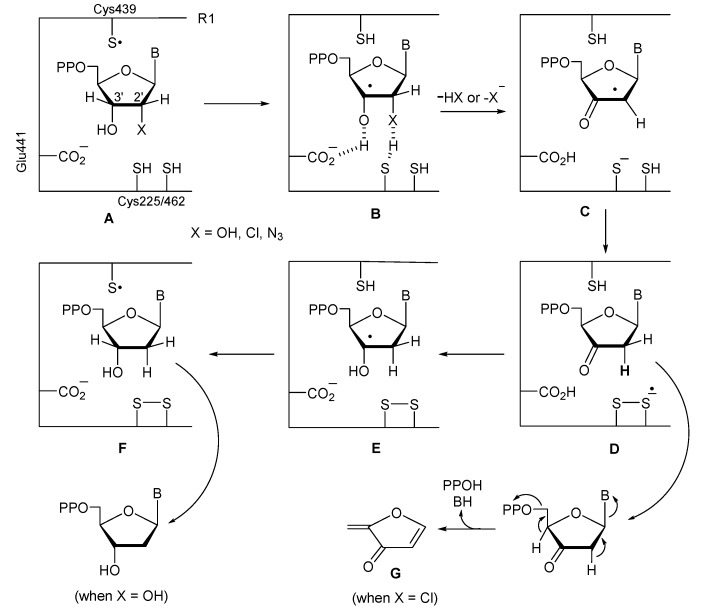
Mechanisms proposed for reduction of ribonucleoside diphosphates and inactivation of RDPRs by 2′-chloro analogues [1].

Abstraction of H3′ from the ribosyl moiety of the substrate in **A** (Figure 1, X = OH) by a thiyl radical (•SCys439) to give **B** (X = OH) is the postulated initial step of the RNR-catalyzed deoxygenation of ribonucleotides [1]. Enzyme-assisted loss of water from C2′ in **B** would produce the 3′-keto C2′ radical in **C**, and further electron and hydrogen-atom transfers via **D** and **E** would complete the conversion of substrate into the product 2′-deoxynucleotide in **F** [11,12].

Abstraction of H3′ from a 2′-chloro-2′-deoxynucleotide inactivator was proposed to convert **A** (X = Cl) to **B** (X = Cl). Spontaneous loss of chloride in **B** (X = Cl) followed by hydrogen transfer from the thiol to C2′ in **C** and electron transfer to the dithiol would produce the identical 3′-keto-2′-deoxynucleotide in **D** as with the substrate nucleotide [1,4,6]. Changes at the active site caused by the loss of Cl^−^ and H^+^ from the inactivator (rather than enzyme-assisted removal of H_2_O from the substrate) were invoked to rationalize its dissociation from **D** of the enzyme. Successive β-eliminations of B(ase) and pyrophosphate would generate 2-methylene-3(2*H*)-furanone (**G**), a Michael acceptor, which could affect covalent alkylating of the RNR and cause mechanism-based inactivation. Incubation of gemcitabine 5′-diphosphate with RDPRs (ribonucleoside diphosphate reductases) caused inactivation of both of the R1 and R2 subunits [13,14,15,16,17].

The initial mechanistic steps are compatible with theoretical modeling studies [18,19], with the biomimetic chemical experiments reported by Giese [20] and Robins [21,22], and with Stubbe’s enzymatic studies with gemcitabine [13,14,15,16,17] and 2′-deoxy-2′-fluoromethylenecytidine [23,24]. However, the detection of ribosyl-based radicals during RNR-catalyzed deoxygenation of substrates remained elusive [25]. Two sets of EPR signals were observed during kinetic studies with the E441Q mutant of *E. coli* class I RDPR and cytidine 5′-diphosphate [26]. Signals attributed to the initially detected radical were consistent with those from a disulfide radical anion [27], and those from the second were compatible with appearance of a nucleotide-based radical of the semidione type [28]. The latter-type of signals was observed during the inactivation of RNR by gemcitabine nucleotides [16].

Giese [20] and Robins [21,22] designed chemical models to simulate such initiation/elimination cascades that begin with the generation of a C3′ radical during the reduction and mechanism-based inactivation mediated by RDPRs. Lenz and Giese employed photochemical fragmentation of adenosine 3′-selenoesters in aqueous methanol to generate C3′ radicals containing a 3′-hydroxyl group. The addition of an acetate buffer to the photolysis solution enhanced the rate of cleavage of water from C2′ as predicted for base-promoted assistance by an enzymatic carboxylate group [20]. Robins et al. designed 6′-*O*-nitro-2′-substituted homonucleoside derivatives that produced 6′-oxyl radicals upon treatment with tributylstannane/AIBN. The O6′ radicals were positioned to abstract H3′ with the generation of C3′ radicals containing a 3′-hydroxyl group. Fragmentation of a 2′-*O*-tosyl derivative occurred by anionic elimination [29], whereas a 2′-chloro analogue fragmented by a radical elimination pathway [30]. Nucleoside derivatives with a 2′-(azido, bromo, chloro, iodo, or methylthio) group also underwent elimination of a radical species upon generation of a radical center at C3′ [31].

Begley recently reported trapping of intermediates in the MoaA-catalyzed biosynthesis of molybdopterin using 2′-deoxyguanosine 5′-triphosphate and its 2′-chloro-2′-deoxy derivative as alternative substrate analogs [32]. A 5′-deoxyadenosyl radical cofactor abstracts H3′ from the GTP substrate (and also from the 2′-chloro ribo analog) with generation of C3′ radicals containing a 3′-hydroxyl group. The homolytic elimination of chlorine from C2′ would give the 2′,3′-enol, and β-eliminations of triphosphate and guanine would produce 2-methylene-3(2*H*)furanone (**G**, Figure 1) as described by us in 1996 [30].

Herein, we report that 1,5-dideoxyhomoribofuranose derivatives containing a 3-hydroxyl group with a bromo, chloro, or tosylate substituent at C2 fragment with loss of bromide, chloride, or tosylate anions upon generation of a radical center at C3. The ionic fragmentations of both ribo and arabino epimers were essentially equivalent. These model reactions provide additional experimental data that allow further illumination of mechanisms employed by ribonucleotide reductases [18,19] and the MoaA enzyme [32] involved in molybdopterin biosynthesis.

## 2. Results

### 2.1. Synthesis of 1,4-Anhydrohexitol Models

**Scheme 1:**
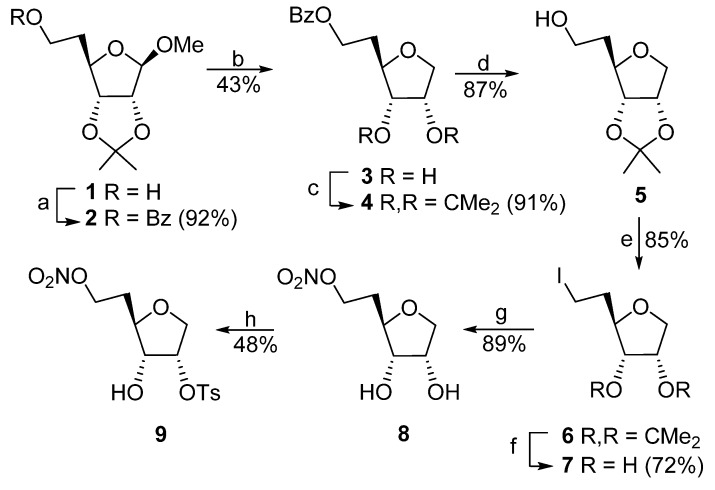
Reagents and Conditions: (a) BzCl/Et_3_N/CH_2_Cl_2_/0 °C; (b) Et_3_SiH (3 equiv)/BF_3_•OEt_2_/CH_2_Cl_2_; (c) TsOH/acetone/2,2-dimethoxypropane; (d) KOH/MeOH; (e) Ph_3_P/I_2_/imidazole/toluene/Δ; (f) HCl/H_2_O/MeOH; (g) AgNO_3_ (2 equiv)/CH_3_CN/rt/48 h; (h) Bu_2_SnO/TsCl (6 equiv)/Et_3_N/MeOH.

Methyl 5-deoxy-2,3-*O*-isopropylidene-β-D-*ribo*-hexofuranoside (**1**, Scheme 1) was prepared efficiently in 7 steps (~40% overall yield) from 1,2-*O*-isopropylidene-α-D-glucose [29]. Benzoylation of **1** and anomeric deoxygenation of **2** (BF_3_•OEt_2_/Et_3_SiH) [33,34] gave a mixture of **4** and **3** (partial loss of the isopropylidene group). Reprotection of **3** gave the 1,4-anhydroalditol [35] **4** (44% overall from **1**). Successive debenzoylation of **4**, iodination of **5**, and deprotection of **6** gave 6-iodo furanitol **7** (53% from **4**). Nitrate displacement of iodide from **7** (AgNO_3_/CH_3_CN) gave 1,4-anhydro-5-deoxy-6-*O*-nitro-D-*ribo*-hexitol (**8**, 89%). Tin-mediated tosylation [36] of **8** gave a regioisomeric mixture from which the major 2-*O*-tosyl isomer **9** (48%) was isolated.

**Scheme 2:**
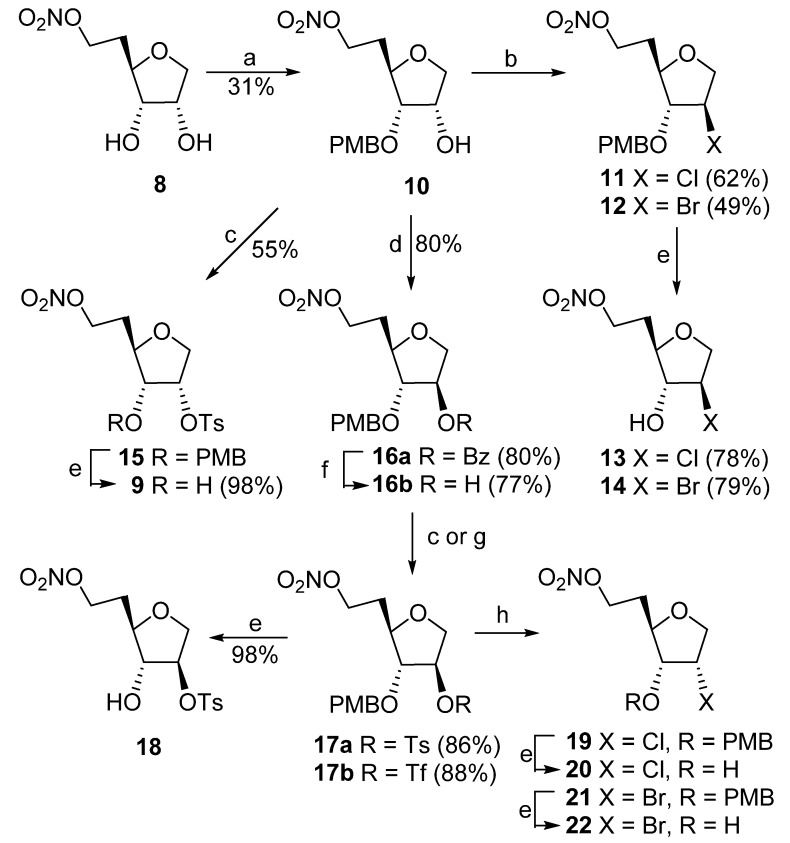
Reagents and Conditions: (a) Bu_2_SnO (1.1 equiv)/PMBCl (2 equiv)/DMF/Δ; (b) Ph_3_P/DIAD/pyridine•HCl (3 equiv) or pyridine•HBr (1.5 equiv)/THF/rt/22 h; (c) TsCl/pyridine/rt/16 h; (d) Ph_3_P/DIAD/BzOH/THF/−50 °C; (e) CAN (3 equiv)/MeCN/H_2_O/rt/22 h; (f) KOH/MeOH; (g) TfCl (1.2 equiv)/DMAP (2.5 equiv)/CH_2_Cl_2_/0 ˚C/1 h; (h) LiCl (5 equiv/rt/5 h) or LiBr (5.5 equiv/rt/7 h)/DMF.

Tin-mediated benzylation of **8** with *p*-methoxybenzyl (PMB) chloride gave a mixture from which the 3-*O*-PMB regioisomer **10** (Scheme 2) was isolated. Tosylation of **10** followed by removal of the PMB group from **15** (ceric ammonium nitrate (CAN)) gave an alternative route to **9** (54% from **10**). Mitsunobu chlorination of **10** (freshly prepared HCl•pyridine) gave the protected 2-chloro-*arabino*-hexofuranose **11** (62%). Analogous bromination of **10** with freshly prepared HBr•pyridine gave the 2-bromo product **12** (49%)**.** Debenzylation of **11** and **12** (CAN) gave **13** (78%) and **14** (79%), respectively.

The 2-chloro and 2-bromo-D-*ribo*-hexofuranoses **20** and **22** (Scheme 2) were prepared from **10** by double inversion. Mitsunobu treatment of **10** (BzOH) produced the 2-*O*-benzoyl-D-*arabino*-hexofuranose **16a.** Debenzoylation of **16a** and treatment of **16b** with TfCl/DMAP gave the reasonably stable triflate **17b**. Displacement of triflate (LiCl or LiBr) and debenzylation (CAN) of the resulting **19** or **21** gave the 2-chloro or 2-bromo-D-*ribo*-hexofuranose **20** or **22**. Tosylation of **16b** followed by debenzylation of **17a** gave the 2-*O*-tosyl-*arabino* product **18**.

**Scheme 3:**
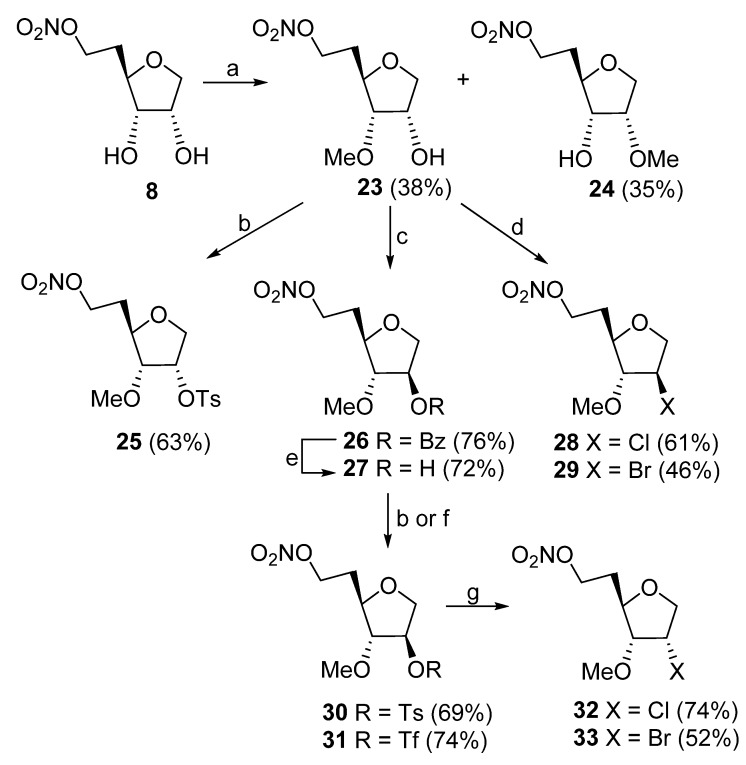
Reagents and Conditions: (a) Bu_2_SnO/MeI/DMF/Δ; (b). TsCl/pyridine; (c) Ph_3_P/DIAD/BzCl/THF/50 °C; (d) Ph_3_P/DIAD/pyridine•HCl or pyridine•HBr (1.5 equiv)/THF/rt/18 h; (e) KOH/MeOH; (f) TfCl/DMAP/CH_2_Cl_2_/0 °C; (g) LiCl (5 equiv) or LiBr (3 equiv)/DMF/rt/5 h.

Tin-mediated methylation of **8** produced **23** and **24** (Scheme 3) and the major 3-*O*-methyl isomer **23** (38%) was isolated and tosylated to give the *ribo* tosylate **25**. Mitsunobu treatment of **23** (BzOH), debenzoylation of **26**, and tosylation of **27** gave *arabino* tosylate **30**. Mitsunobu halogenations of **23** gave the 3-*O*-methyl-D-*arabino* 2-chloro **28** and 2-bromo **29** analogues. Triflation of **27** and displacement of triflate from **31** with LiCl or LiBr gave the *ribo* 2-chloro **32** and 2-bromo **33** compounds.

### 2.2. Radical Generation Studies

**Scheme 4:**
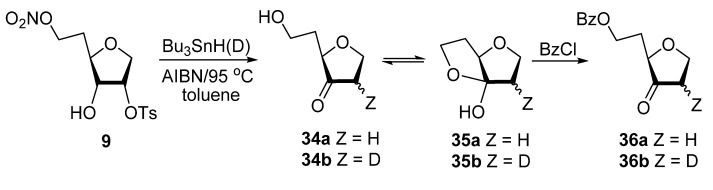
Biomimetic studies with 6-*O*-nitro-1,4-anhydrohexitols.

Treatment of the 6-*O*-nitro-2-*O*-tosyl *ribo* compound **9** with Bu_3_SnH/AIBN/toluene/95 °C for 1 h gave an equilibrating mixture of the 3-oxo product **34a** [(*R*)-2-(2-hydroxyethyl)-3(2*H*)-dihydrofuranone] and its cyclic hemiacetal **35a** (66%, ~1:1; ^1^H and ^13^C NMR) (Scheme 4). A minor amount (19%) of the 6-hydroxy-2-*O*-tosyl byproduct resulting from hydrogen transfer to the 6-oxyl radical without elimination of tosylate also was isolated (Table 1, entry 1, footnote d). The ^13^C NMR spectrum of the mixture had a signal for the carbonyl carbon of **34a** at 215.9 ppm and one for the hemiacetal carbon of **35a** at 114.9 ppm. Analogous treatment of **9** with Bu_3_SnD gave the 2-deuterio epimers (2*R/S*, ~1:1) of **34b**/**35b** (71%, ~1:1; entry 2). The ^1^H NMR spectrum of **34b**/**35b** corresponded to that of **34a**/**35a** with ~50% reduction of the integrated intensity for the H2/2′ signals and simplification of the H1/1′ signals. The ^13^C NMR spectrum of **34b**/**35b** showed triplets from splitting with deuterium (1:1:1 intensity) at 36.7 and 38.9 ppm for C2. MS and HRMS spectra showed ~95% incorporation of deuterium. Benzoylation of the **34a/35a** mixture provided a stable ketone benzoate **36a**, and benzoylation of **34b/35b** gave **36b** (2-deuterio epimers).

**Scheme 5:**
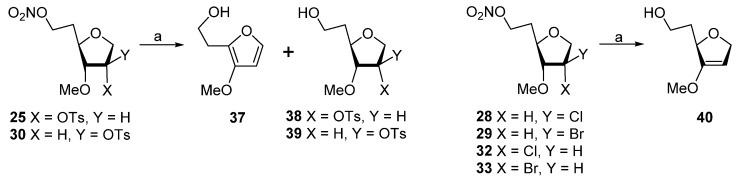
Biomimetic studies with 3-*O*-methyl-6-*O*-nitro-1,4-anhydrohexitols. Reagents and Conditions: (a) Bu_3_SnH(D)/AIBN/toluene/95 °C.

The role of the proton on the 3-hydroxyl group was probed. Treatment of the 3-*O*-methyl-6-*O*-nitro-2-*O*-tosyl ribo substrate **25** produced 2-(2-hydroxyethyl)-3-methoxyfuran (**37**, 63%) plus the 6-hydroxy byproduct **38** (22%) (Scheme 5; Table 2, entry 1). Treatment of **25** with Bu_3_SnD gave **37** and **38** with no incorporation of deuterium (entry 2) and no epimerization at C3 [37]. Tosylate **25** was thermally stable in toluene at 95 °C. Treatment of the arabino 3-*O*-methyl-2-*O*-tosyl substrate **30** with Bu_3_SnH gave **37** (51%) plus byproduct **39** (38%) (entry 7). Treatment of **30** with Bu_3_SnD produced **37** and **39** without incorporation of deuterium or epimerization at C3 (entry 8).

Treatment of the 3-*O*-methyl-2-chloro (**32**) and 2-bromo (**33**) ribo substrates with Bu_3_SnD resulted in formation of vinyl ether **40** with no incorporation of deuterium (entries 3 and 4). The ^13^C NMR spectrum of **40** had olefinic signals at 90.2 and 157.7 ppm, and the ^1^H NMR spectrum had olefinic proton signals at 4.60–4.70 ppm. Treatment of the arabino 3-*O*-methyl-2-chloro (**28**) and bromo (**29**) compounds with Bu_3_SnD also resulted in elimination of a halogen atom to give vinyl ether **40** with no incorporation of deuterium (entries 5 and 6).

Similar treatment of the 2-chloro (**20**) and 2-bromo (**22**) ribo analogs produced the same keto/hemiacetal mixture (**34a**/**35a**, ~1:1; 81%, entry 3; and 64%, entry 5). Treatment of **20** (and **22**) with Bu_3_SnD gave **34b**/**35b** as 2(*R*/*S*)-deuterio epimers (entries 4 and 6). Toluene solutions of **9**, **20**, and **22** (without Bu_3_SnH or AIBN) were heated independently at 95 °C for 4 h (also at 110 °C). Unchanged starting materials were recovered almost quantitatively, which confirmed their thermal stability under these conditions and excluded the possibility of initial dissociation of a substituent from C2. Compound **9** also was stable at 95 °C for 2.5 h in DMF.

The arabino 2-(chloro, bromo, and tosyloxy) compounds (**13**, **14**, and **18**) were subjected to the conditions used with the ribo epimers to probe the effect of stereochemical inversion at C2. The same equilibrating mixtures of **34a**/**35a** were produced with Bu_3_SnH (entries 7, 9, and 11) and the epimeric 2-deuterio derivatives **34b**/**35b** were generated with Bu_3_SnD (entries 8, 10, and 12). Incorporation of deuterium into the ketone/hemiacetal products demonstrated that elimination of tosylate, chloride, and bromide anions occurred with all three of the arabino compounds. Enhanced yields of the tautomeric product mixtures were isolated with the chloro (**13**, entries 7 and 8) and tosylate (**18**, entries 11 and 12) substrates. Byproduct formation was not observed with **18**, whereas it was formed (~19%) with the ribo substrate **9** (entries 1 and 2, footnote d). Some hydrodebromination also was detected with the arabino 2-bromo epimer **14** (entries 9 and 10, footnote e).

**Figure 2:**
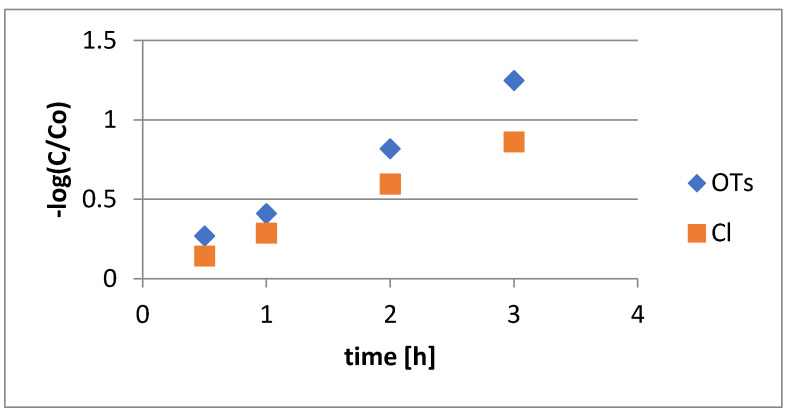
Pseudo-first order plots for conversion of **13** (chloro) and **18** (tosylate) into **34a/35a.**

Samples of **9**, **13**, **14**, and **18** were treated with Bu_3_SnH in deuterated toluene. Fragmentation of tosylate **9** in toluene-*d*_8_ (Bu_3_SnH/AIBN) at 75 °C was 90–95% complete in 2.5 h (TLC and ^1^H NMR) and produced **34a/35a** (80–85%) as sole products with no incorporation of deuterium. Analogous treatment of **9** at 55 °C for 2.5 h resulted in ~50% fragmentation. Treatment of the arabino tosylate **18** and chloride **13** substrates at 75 °C showed that the fragmentation of **18** was slightly faster than that of **13** (Figure 2; ^1^H NMR spectra were used for substrate/products ratios, see the Experimental Section). Fragmentation of the ribo tosylate **9** to give **34a/35a** proceeded at a rate similar to that determined with the arabino chloride **13**. Treatment of the arabino bromide **14** at 75 °C produced **34a/35a** plus 2-deoxy byproduct mixtures with ^1^H NMR spectra that were too complex for quantitative analysis.

## 3. Discussion

The mechanism proposed [1] for conversion of ribonucleoside 5′-diphosphates (**A**, X = OH) to 2′-deoxynucleotides (**F**) by RDPRs (Figure 1, inside the boxes) is supported by biochemical, chemical, and theoretical modeling studies. However, the enzymatic processing of 2′-chloro analogs (**A**, X = Cl) could cause inactivation by different chemistry. Incubation of a 2′-chloro-2′-deoxynucleoside 5′-diphosphate with RDPR produced 2-methylene-3(2*H*)furanone (**G**), a Michael acceptor that could cause time-dependent enzyme inactivation. Stubbe rationalized [1] that spontaneous elimination of a chloride anion (and a proton) at the active site (rather than the enzyme-assisted removal of HOH with substrates) could cause active site changes resulting in dissociation of the 2′-deoxy-3′-oxo intermediate from **D** (Figure 1). Successive β-eliminations of pyrophosphate from C5′ and the base from C1′ could generate **G** in solution. However, the identical 3′-keto intermediate in **D** was postulated in the substrate to product sequence, which makes the presence of chloride and a proton the only difference for the inactivation sequence.

We reasoned that elimination of a chlorine atom from the initial C3′ radical was more likely. The electronegative character of C1′ would make the elimination of chloride (with generation of positive character at C2′) unfavorable, whereas loss of a chlorine atom with generation of a C2′ radical was well precedented [38], and generation of an enol would be energetically advantageous. Elimination of a chlorine atom (rather than chloride) at the active site could have serious consequences. The chlorine radical could attack a sulfhydryl group and the resulting sulfenyl chloride could react with nucleophilic groups in the enzyme or undergo hydrolysis to a sulfenic acid. Chlorine-atom abstraction of hydrogen from an amino acid residue (and resulting radical processes) and other chlorine-radical reactions would be possible, whereas such events would not occur with a ground state chloride anion. Radical-induced disruption of active-site architecture provides a more plausible explanation for dissociation of the 2′-deoxy-3′-oxo intermediate from **D**.

**Figure 3:**
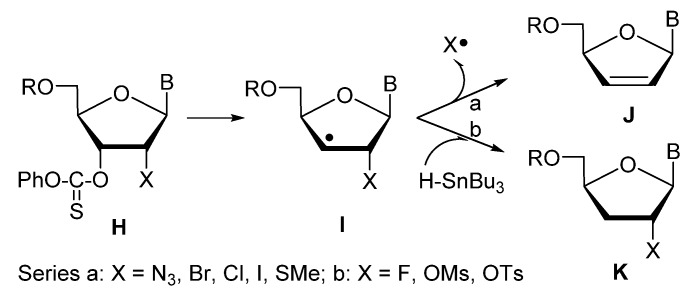
Bu_3_SnH/AIBN/Δ treatment of 3′-thionocarbonates.

We have shown [31] that leaving-group radical stability is crucial for substituent elimination from C2′ upon generation of a radical at C3′. Treatment of 2′-(azido, bromo, chloro, iodo, or methylthio)-2′-deoxy-3′-*O*-phenoxythiocarbonyl nucleosides (**H**a, Figure 3) with BuSnH resulted in generation of C3′ radicals **I**a that underwent elimination of a radical from C2′ with formation of 2′,3′-unsaturated derivative **J**. In contrast, treatment of the 2′-(fluoro, mesyloxy, or tosyloxy)-3′-thionocarbonates **H**b generated C3′ radicals **I**b that abstracted hydrogen from the stannane to give 3′-deoxy-2′-(fluoro, mesyloxy, or tosyloxy) derivatives **K**b. Elimination of a radical from C2′ of **I**a gave **J**, whereas homolytic scission to release a high-energy fluorine atom or a mesyloxy or tosyloxy radical is energetically prohibitive—and did not occur. In those cases, abstraction of hydrogen from the stannane by the C3′ radical **I**b gave the reduced products **K**b.

**Figure 4:**
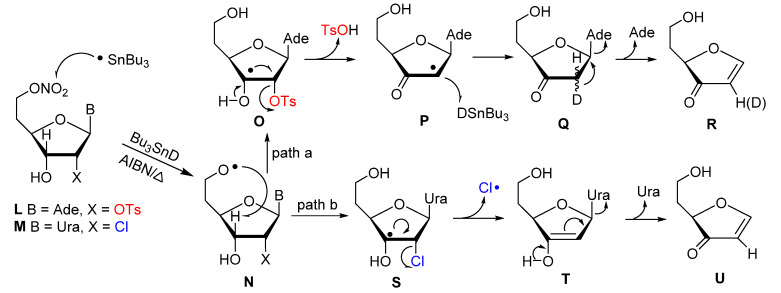
Heterolytic (path a, TsO^−^) and homolytic (path b, Cl•) eliminations from radicals containing a 3′j32;-hydroxyl group [21,29,30].

Because the radicals **I** did not contain a 3′-hydroxyl group, we prepared model compounds that were more closely related to the natural substrates for RDPR-catalyzed 2′-deoxygenations. Treatment of the adenine 6′-*O*-nitro-2′-*O*-tosyl **L** and uracil 6′-*O*-nitro-2′-chloro **M** analogues with Bu_3_SnD/AIBN/Δ generated the 6′-oxyl radicals **N** by attack of a stannyl radical at nitrate oxygen (Figure 4). Intramolecular abstraction of H3′ from **N** gave the C3′ radicals **O** or **S**. Loss of the proton from the 3′-hydroxyl group of **O** and elimination of the 2′-tosylate (with shift of the unpaired electron from C3′ to C2′ and generation of the O = C3′ double bond) would drive the overall elimination of toluenesulfonic acid from **O** to produce **P**. Deuterium transfer from the stannane to C2′ of **P** followed by elimination of the trans proton/deuteron and adenine from **Q** would give the observed 2-(2-hydroxyethyl)-3(2*H*)-furanone (**R**) containing deuterium at C4 (C2′ from the nucleoside). In contrast, loss of the chlorine atom from **S** would give enol **T**, which could undergo 1,4-elimination to **U** with no incorporation of deuterium (as observed). This demonstrated the distinct differences between TsO–C2′ bond cleavage by a two-electron heterolysis (in **O**) and cleavage of the chlorine–C2′ bond by a one-electron homolysis (in **S**) [21,29,30].

Barton’s nitrite ester [39] and Wagner’s *δ*-substituted aryl ketone [40] photolysis studies showed a strong preference for six-membered transition states for hydrogen abstraction by oxyl radicals. Fraser-Reid employed that [1,5]-hydrogen shift with oxyl radicals generated from carbohydrate nitrate esters and Bu_3_SnH [41]. Radical-induced loss of **^–^**OTs and H^+^ (Figure 4, path a) is also analogous to an ionic LiEt_3_BH-promoted rearrangement of 2′-*O*-tosyladenosine. Removal of the 3′-hydroxyl proton by Et_3_BH^–^ initiated a [1,2]-hydride shift from C3′ to C2′ with displacement of tosylate from C2′ [42,43]. That rearrangement occurred also with 2′-chloro-2′-deoxyadenosine, but at a lower rate with the poorer leaving group (chloride).

Theoretical modeling [18,44] of RDPR-catalyzed 2′-deoxygenation invoked hydrogen bonding from the 3′-OH to a carboxylate group and from H-donors to 2′-OH. Analogous attraction between the cis 3-OH in **9** and a tosylate oxygen atom was considered for possible assistance of the heterolytic cleavage of the TsO–C2 linkage. However, treatment of the arabino tosylate **18** (no cis 3-OH) gave the same **34b**/**35b** mixture in higher yield (93%) than with **9** (~70%). Byproduct with a hydroxyl group at C6 and tosylate at C2 was isolated (~19%) from treatment of **9**, but no such arabino byproduct was observed with **18**. These results are more consistent with a greater population of the C2-endo/C3-exo conformation range in **9** (reduction of strain with the 2-tosylate group) that would make a six-membered transition state for intramolecular abstraction of H3 by the 6-oxyl radical less favorable. Enhanced abstraction of deuterium from the stannane by the 6-oxyl radical would then occur to produce more of the byproduct. Greater C2-exo/C3-endo populations in **18** (reduction of strain between the arabino tosylate at C2 and the side chain at C4) would enhance the approach of the 6-oxyl radical for H3 abstraction and elimination of tosylate from the C3 radical to produce **34b**/**35b** in higher yield (93%).

Thus, noteworthy mechanistic changes were observed within our model series. Anionic elimination of tosylate from C2′ of a nucleoside occurred (Figure 4, pathway a), whereas radical elimination of a chlorine atom (pathway b) was preferred. However, treatment of our ribo anhydroalditol tosylate **9**, chloro **20**, or bromo **22** substrates produced the same 2-deuterio-3-ketone **34b** and hemiacetal **35b** mixture. The arabino chloro **13** and bromo **14** epimers also gave **34b**/**35b** (plus some hydrodebromination with **14**). In every case, the loss of bromide or chloride anions from an intermediate C3 radical resulted in formation of deuterium-labeled materials rather than homolytic loss of a halogen atom to give the unlabeled alkene. The absence of an electronegative nucleobase on C1 (C1 is a –CH_2_– moiety in the anhydroalditol models) allowed elimination of an anion from C2 when loss of the 3-hydroxyl proton could generate an O = C3 double bond.

Radical elimination occurred with the 2-chloro-3-*O*-methyl ribo **32** and arabino **28** substrates to give vinyl ether **40**. Both 2-bromo-3-*O*-methyl ribo **33** and arabino **29** epimers also gave **40** (plus hydrodebrominated byproduct). Thus, when loss of the 3-hydroxyl proton and generation of a 3-ketone was prevented, elimination of a halogen atom occurred.

Ramos questioned our choice of toluene as solvent and lack of a basic residue in our prior nucleoside model studies and stated: “we concluded that the nature of the leaving substituent can be controlled; it will be anionic or radical depending on the presence or absence of a basic residue capable of deprotonating the 3′-HO group. In the enzyme, such functionality does exist, and so it can be concluded that the enzyme indeed eliminates anions, and not radicals” [19].

Clearly, the elimination of an anion is possible without a basic residue present in our current anhydroalditol models. Siegbahn [18] calculated a dielectric constant of ε ~ 4 in the region of the enzyme active site, which is approximated much more closely by that of toluene (ε ~ 2.4) than that of the aqueous methanol solutions (ε > 40) used by Lenz and Giese [20]. Thus, the intrinsic electronic status of the anomeric carbon C1′ (inductively negative in nucleosides) or C1 (inductively positive in anhydroalditols) as well as the nature of the leaving substituent (X• or A^−^) are major factors that control heterolytic elimination of an anion or homolytic elimination of a radical in biomimetic model reactions—and such factors also likely play a key role in enzyme-initiated inactivation processes.

Begley [32] also has invoked our elimination of a chlorine atom [30] upon abstraction of H3′ from 2′-chloro-2′-deoxyguanosine triphosphate by a 5′-deoxyadenosyl radical cofactor in MoaA. His 2′-deoxy-3′-ketone underwent the sequential β-eliminations [30] of triphosphate and guanine to give the same 2-methylene-3(2*H*)furanone (**G**, Figure 1) that could cause inactivation of RDPRs. Both of our ribo **32** and arabino **28** 2-chloro epimers gave vinyl ether **40**, whereas Begley’s arabino epimer remained unchanged during incubation with MoaA. The cis,cis configuration of H3′/Cl/guanine in his arabino isomer might restrict conformational ranges that would favor binding with MoaA, and/or hinder approach of the 5′-deoxyadenosyl cofactor toward H3′. As discussed above, the different product/byproduct ratios in our fragmentations of **9** and **18**, and **25** and **30** might result from such conformational effects.

**Figure 5:**
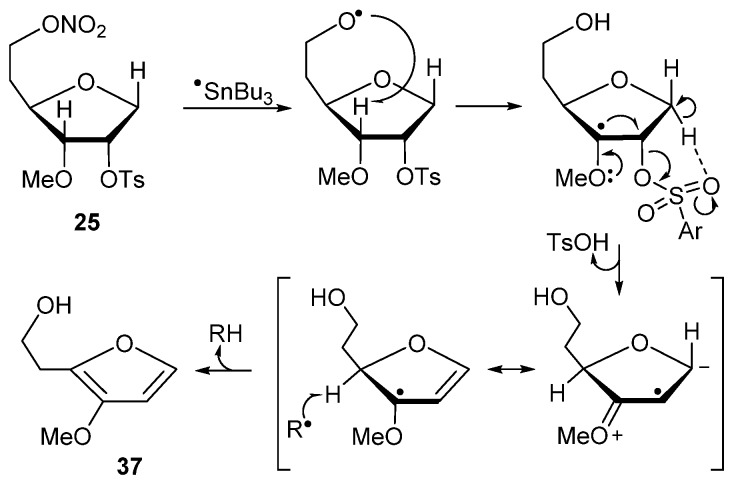
TsOH elimination and aromatization.

Treatment of the 3-*O*-methyl-2-*O*-tosyl ribo **25** or arabino **30** substrates produced the aromatized 2-(2-hydroxyethyl)-3-methoxyfuran (**37**) plus the respective 6-hydroxy-2-*O*-tosyl byproducts **38** or **39**. Generation of a carbonyl group at C3 is precluded with **25** and **30**, but a favorable six-membered transition state involving attraction between the α-proton on C1 and a tosylate oxygen with epimer **25** (Figure 5) might aid the loss of HOTs with generation of a 1,2-double bond. An analogous interaction involving the β-proton on C1 and a tosylate oxygen with arabino epimer **30** also is possible. Abstraction of hydrogen from C4 of the resulting resonance hybrid would produce furan **37**. The observed similar yields of **37** plus the respective 2-*O*-tosyl byproducts **38** and **39** are consistent with parallel processes for the ribo **25** and arabino **30** epimers. Tosylate **25** was stable in toluene at 95 °C, which confirmed that generation of a C3 radical was necessary for the elimination of tosylate and production of **37**.

## 4. Experimental Section

The ^1^H (400 or 500 MHz) and ^13^C (100 or 125 MHz) NMR spectra were determined on Bruker Biospin spectrometers (Bruker, Billerica, MA, USA) with solutions in CDCl_3_ unless otherwise noted. HRMS were obtained in TOF-ESI mode on Bruker Solarix FT-ICR instrument (Bruker, Billerica, MA, USA) unless otherwise noted. TLC was performed with Merck (Darmstadt, Germany) kieselgel 60-F_254_ sheets and products were detected with 254 nm light or by visualization with Ce(SO_4_)_2_/(NH_4_)_6_Mo_7_O_24_∙4H_2_O/H_2_SO_4_/H_2_O reagent. Merck kieselgel 60 (230–400 mesh) was used for column chromatography. Reagent grade chemicals were used, and solvents were dried by reflux over and distillation from CaH_2_ (except for THF/potassium) under argon or by passing the solvents through activated alumina cartridges using a solvent purification system.

**Methyl 6-*O*-Benzoyl-1,5-dideoxy-2,3-*O*-isopropylidene-β-D-*ribo*-hexofuranoside (2).** To a solution of methyl 1,5-dideoxy-2,3-isopropylidene-β-D-*ribo*-hexofuranoside [29] (**1**; 13.0 g, 58.8 mmol) and Et_3_N (8.9 g, 12.3 mL, 88.2 mol) in CH_2_Cl_2_ (50 mL) was added dropwise BzCl (12.4 g, 10.2 mL, 88.2 mmol) at 0 °C. The cooling bath was removed and after 1 h the reaction mixture was quenched by addition of MeOH (2 mL). After additional 30 min the volatiles were evaporated and the residue was column chromatographed (hexanes → hexanes/EtOAc, 6:1) to give **2** as pale yellow oil (17.3 g, 92%): ^1^H NMR δ 1.32, 1.49 (2 × s, 2 × 3H), 2.01–2.04 (m, 2H), 3.38 (s, 3H), 4.39–4.52 (m, 3H), 4.62–4.65 (m, 2H), 4.98 (s, 1H), 7.42–7.57 (m, 3H), 8.04–8.06 (m, 2H); ^13^C NMR δ 24.6, 26.2, 33.9, 54.8, 61.6, 83.6, 84.0, 85.2, 109.6, 112.0, 128.1, 129.3, 129.9, 132.6, 166.0; MS FAB *m*/*z* 345 (3, [M + Na]^+^), 291 (100, [M − OMe]^+^); HRMS ESI *m*/*z* calcd for C_17_H_22_O_6_Na [M + Na]^+^ 345.1314, found 345.1317.

**6-*O*-Benzoyl-1,5-dideoxy-D-*ribo*-hexofuranose** (**3)** and **6-*O*-Benzoyl-1,5-dideoxy-2,3-*O-* isopropylidene-D-*ribo*-hexofuranose (4).** To a stirred solution of **2** (18.0 g, 55.9 mmol) and Et_3_SiH (19.4 g, 26.7 mL, 167.7 mmol) in CH_2_Cl_2_ (10 mL) was added BF_3_•OEt_2_ (23.8 g, 21.3 mL, 167.7 mmol). Mildly exothermic reaction ensued after approx. 15 min and the reaction mixture was allowed to stir for an additional 3 h. The reaction flask was placed in an ice slush and a saturated NaHCO_3_/H_2_O solution (200 mL) was added slowly with vigorous stirring. CH_2_Cl_2_ was added (50 mL), aqueous phase was separated and washed with CH_2_Cl_2_ (50 mL). Organic fractions were combined and evaporated to dryness. Column chromatography (hexanes/EtOAc, 6:1 → EtOAc) gave contaminated (~10%) **4** (1.5 g, 9%) and **3** (6.1 g, 43%) as syrup. Compound **3** had: ^1^H NMR (CD_3_OD) δ 1.87–1.94 (m, 1H), 2.12–2.19 (m, 1H), 3.70 (dd, *J* = 2.9, 9.8 Hz, 1H), 3.79 (dd, *J* = 5.4, 7.3 Hz, 1H), 3.85–3.89 (m, 1H), 4.07 (dd, *J* = 4.9, 9.8 Hz, 1H), 4.16 (td, *J* = 2.9, 4.9 Hz, 1H), 4.38–4.50 (m, 2H), 7.45–7.61 (m, 3H), 8.02–8.04 (m, 2H); ^13^C NMR (CD_3_OD) δ 32.4, 61.9, 70.8, 72.2, 75.9, 78.9, 128.3, 129.5, 129.9, 133.0, 166.8; MS FAB *m*/*z* 275 (100, [M + Na]^+^); HRMS ESI *m*/*z* calcd for C_13_H_16_O_5_Na [M + Na]^+^ 275.1531, found 275.0902.

Diol **3** (6.1 g, 24.2 mmol) and TsOH hydrate (0.5 g, 2.6 mmol) were dissolved in a mixture of acetone (40 mL) and 2,2-dimethoxypropane (10 mL) and left to stir at room temperature for 30 min. Neutralization with sat. NaHCO_3_ (100 mL) followed by EtOAc extraction (2 × 50 mL) and evaporation to dryness provided oil that was filtered through silica to give **4** (6.4 g, 91%): ^1^H NMR δ 1.33, 1.52 (2 × s, 2 × 3H), 1.86–1.92 (m, 2H), 3.86 (dd, *J* = 4.4, 10.7 Hz, 1H), 3.97 (dd, *J* = 1.5, 10.7 Hz, 1H), 4.23–4.26 (m, 1H), 4.36–4.49 (m, 2H), 4.52 (dd, *J* = 1.5, 6.3 Hz, 1H), 4.80–4.83 (m, 1H), 7.42–7.57 (m, 3H), 8.03–8.05 (m, 2H); ^13^C NMR δ 24.9, 26.5, 29.7, 61.5, 71.6, 80.8, 81.2, 85.0, 112.8, 128.3, 129.5, 130.1, 132.9, 166.4. MS FAB *m*/*z* 293 (100, [M + H]^+^), 315 (15, [M + Na]^+^); HRMS ESI *m*/*z* calcd for C_16_H_20_O_5_Na [M + Na]^+^ 315.1208, found 315.1209.

**1,5-Dideoxy-2,3-*O*-isopropylidene-D-*ribo*-hexofuranose (5).** To a stirred solution of **4** (14.0 g, 47.9 mmol) in MeOH (50 mL) was added a solution of KOH (2.0 g, 35.7 mol) in MeOH (50 mL). After 1 h the volatiles were evaporated and the residue was column chromatographed (hexanes/EtOAc, 2:1 → EtOAc) to give **5** [45] (8.0 g, 87%): ^1^H NMR δ 1.33, 1.51 (2 × s, 2 × 3H), 1.63–1.76 (m, 2H), 3.74–3.82 (m, 2H), 3.89 (dd, *J* = 4.4, 10.4 Hz, 1H), 3.94 (d, *J* = 10.3 Hz, 1H), 4.19–4.22 (m, 2H), 4.48 (dd, *J* = 1.5, 5.8 Hz, 1H), 4.81 (t, *J* = 5.1 Hz, 1H); ^13^C NMR δ 24.5, 26.2, 32.4, 59.2, 71.1, 80.4, 82.2, 84.6, 112.3. MS FAB *m*/*z* 189 (100, [M + H]^+^); HRMS ESI *m*/z calcd for C_9_H_17_O_4_ [M + H]^+^ 189.1127, found 189.1123.

**6-Iodo-1,5,6-trideoxy-2,3-*O*-isopropylidene-D-*ribo*-hexofuranose (6).** To a stirred solution of **5** (9.0 g, 47.9 mmol), Ph_3_P (15.1 g, 57.6 mmol) and imidazole (7.9 g, 116.2 mmol) in toluene (225 mL) was added iodine (14.6 g, 57.5 mmol) at 70 °C. The suspension was vigorously stirred another 2 h, then allowed to cool to ambient temperature and the solution decanted to another flask. Volatiles were evaporated and the residue was column chromatographed (hexanes → hexanes/EtOAc, 6:1) to give **6** (13.03 g, 85%): ^1^H NMR δ 1.33, 1.52 (2 × s, 2 × 3H), 1.88–1.97 (m, 2H), 3.14–3.27 (m, 2H), 3.78 (dd, *J* = 4.4, 10.7 Hz, 1H), 3.95 (dd, *J* = 1.5, 10.7 Hz, 1H), 4.10 (ddd, *J* = 1.5, 4.9, 9.2 Hz, 1H), 4.23 (dd, *J* = 2.0, 6.3 Hz, 1H), 4.78 (m, 1H); ^13^C NMR δ 1.0, 24.7, 26.2, 34.0, 71.2, 80.4, 83.6, 84.1, 112.3; MS FAB *m*/*z* 321 (12, [M + Na]^+^), 319 (100); HRMS ESI *m*/*z* calcd for C_9_H_15_IO_3_Na [M + Na]^+^ 320.9964, found 320.9970.

**6-Iodo-1,5,6-trideoxy-D-*ribo*-hexofuranose (7).** A solution of **6** (14.0 g, 47.0 mmol) in a mixture of MeOH (100 mL), H_2_O (25 mL) and conc. HCl (25 mL) was stirred at room temperature for 3 h. Volatiles were evaporated and the residue was column chromatographed (hexanes/EtOAc, 2:1 → EtOAc) to give **7** (8.73 g, 72%): ^1^H NMR δ 2.00–2.07 (m, 1H), 2.16–2.25 (m, 1H), 3.22–3.45 (m, 4H), 3.74–3.77 (m, 2H), 3.84 (br s, 1H), 4.10 (dd, *J* = 4.9, 10.3 Hz, 1H), 4.27 (br s, 1H); ^13^C NMR δ 1.7, 37.3, 70.9, 72.8, 75.6, 81.6; MS FAB *m*/*z* 258 (10, [M]^+^), 74 (100); HRMS ESI *m*/*z* calcd for C_6_H_11_IO_3_, 257.9753, found 257.9740.

**1,5-Dideoxy-6-*O*-nitro-D-*ribo*-hexofuranose (8).** A suspension of **7** (12.0 g, 46.5 mmol) and AgNO_3_ (15.8 g, 93.0 mmol) in CH_3_CN (150 mL) was stirred at room temperature for 2 days. The yellow precipitate was filtered off, washed with EtOAc and the volatiles were evaporated. Column chromatography (hexanes/EtOAc, 1:1 → EtOAc) gave **8** (7.99 g, 89%): ^1^H NMR δ 1.88–1.96 (m, 1H), 2.08–2.15 (m, 1H), 3.43 (br s, 2H), 3.73–3.83 (m, 3H), 4.12 (dd, *J* = 4.9, 10.3 Hz, 1H), 4.23–4.26 (m, 1H), 4.56–4.66 (m, 2H); ^13^C NMR δ 30.2, 70.1, 70.8, 72.6, 75.9, 77.8; MS FAB *m/z* 216 (100, [M + Na]^+^); HRMS ESI *m*/*z* calcd for C_6_H_11_NO_6_Na [M + Na]^+^, 216.0484 found 216.0497.

**1,5-Dideoxy-6-*O*-nitro-2-*O*-tosyl-D-*ribo*-hexofuranose (9). Method A.** A solution of diol **8** (370 mg, 1.92 mmol) and Bu_2_SnO (477 mg, 1.92 mmol) in anhydrous MeOH (40 mL) was heated in a sealed flask at 75 °C for 1 h. After the flask was cooled to 0 °C, Et_3_N (1.15 g, 1.6 mL, 11.4 mmol) was added with stirring followed by TsCl (2.17 g, 11.4 mmol) dissolved in a minimum volume of acetone. The volatiles were evaporated, and the residue was suspended in acetone and deposited on silica. Column chromatography (hexanes/EtOAc, 6:1 → 3:1) gave a 5:2 mixture of two isomers (670 mg) from which the main product **9** (320 mg, 48%) was isolated after second column chromatography: ^1^H NMR δ 1.85–1.92 (m, 1H), 2.09–2.17 (m, 1H), 2.46 (s, 3H), 2.76 (d, *J* = 7.8 Hz, 1H), 3.75 (dt, *J* = 3.9, 8.3 Hz, 1H), 3.80 (dd, *J* = 3.1, 11.2 Hz, 1H), 3.89 (dt, *J* = 5.4, 7.8 Hz, 1H), 4.06 (dd, *J* = 4.9, 11.2 Hz, 1H), 4.52–4.62 (m, 2H), 4.93 (dt, *J* = 3.0, 5.3 Hz, 1H), 7.38, 7.82 (2 × d, *J* = 8.3 Hz, 2 × 2H); ^13^C NMR δ 21.6, 30.2, 69.8, 70.1, 74.9, 77.8, 79.4, 127.8, 130.1, 132.8, 145.6; MS FAB *m*/*z* 370 (100, [M + Na]^+^); HRMS ESI *m*/*z* calcd for C_13_H_17_NO_8_SNa [M + Na]^+^ 370.0573, found 370.0587.

**Method B.** Step a: TsCl (66 mg, 0.35 mmol) was added to a stirred solution of **10** (100 mg, 0.32 mmol) in anhydrous pyridine (1 mL) at ambient temperature. After 16 h, the volatiles were evaporated and residue was partitioned between ice-cold AcOH/H_2_O (1:99, 30 mL) and CHCl_3_ (30 mL). The aqueous layer was extracted with CHCl_3_ and the combined organic phase was washed with ice-cold saturated NaHCO_3_/H_2_O (30 mL), brine (30 mL) and dried (Na_2_SO_4_). Column chromatography (EtOAc/hexane, 5:95 → 35:65) gave 1,5-dideoxy-3-*O*-(*p*-methoxybenzyl)- 6-*O*-nitro-2-*O*-tosyl-D-*ribo*-hexofuranose (**15**; 82 mg, 55%): ^1^H NMR δ 1.78–1.81 (m, 1H), 2.00–2.02 (m, 1H), 2.47 (s, 3H), 3.59 (dd, *J* = 5.0, 8.6 Hz, 1H), 3.83 (s, 3H), 3.86 (dt, *J* = 2.2, 9.6 Hz, 2H), 4.04 (dd, *J* = 4.5, 11.2 Hz, 2H), 4.26 (d, *J* = 11.2 Hz, 1H), 4.47 (m, 2H), 4.58 (d, *J* = 11.2Hz, 1H), 5.13 (dt, *J* = 4.7, 7.0 Hz, 1H), 6.88 and 7.21 (2 × d, *J* = 9.5 Hz, 2 × 2H), 7.34 and 7.84 (2 × d, *J* = 8.3 Hz, 2 × 2H). In step b, A solution of CAN (176 mg, 0.32 mmol) and **15** (75 mg, 0.16 mmol) in MeCN (1.5 mL) and water (0.15 mL) was stirred at ambient temperature for 22 h. Volatiles were evaporated and the residue was column chromatographed (EtOAc/hexane, 3:97 → 30:70) to give **9** (54 mg, 98%) with data as above.

**1,5-Dideoxy-3-*O*-(*p*-methoxybenzyl)-6-*O*-nitro-D-*ribo*-hexofuranose (10).** A suspension of diol **8** (1.20 g, 6.22 mmol) and Bu_2_SnO (1.70 g, 6.84 mmol) in anhydrous MeOH (10 mL) was heated in a sealed flask at 75 °C for 1 h. After the flask was cooled to ambient temperature, the volatiles were evaporated. DMF (5 mL) and PMBCl (1.95 g, 1.7 mL, 12.44) were added, and the reaction mixture was heated at 90 °C for 18 h. Volatiles were evaporated, and the residue was chromatographed (hexanes/EtOAc, 6:1 → 1:1) to give 1,5-dideoxy-2,3-di-*O*-(*p*-methoxybenzyl)-6-*O*-nitro-D-*ribo*- hexofuranose (1.55 g; 20% contaminated) followed by 1,5-dideoxy-2-*O*-(*p*-methoxybenzyl)-6-*O*-nitro-D-*ribo*-hexofuranose (0.47 g, 24%) and **10** (0.60 g; 31%) of sufficient purity for use in subsequent reaction. Sample of **10** was repurified on column chromatography for spectroscopic characterization: ^1^H NMR δ 1.77–1.85 (m, 1H), 1.93–2.00 (m, 1H), 3.20 (br s, 1H), 3.60 (dd, *J* = 5.4, 7.3 Hz, 1H), 3.76 (dd, *J* = 2.9, 10.3 Hz, 1H), 3.81 (s, 3H), 3.85 (dt, *J* = 3.9, 7.3 Hz, 1H), 4.01 (dd, *J* = 4.9, 10.3 Hz, 1H), 4.18 (m, 1H), 4.47–4.61 (m, 4H), 6.88–6.92 (m, 2H), 7.24–7.30 (m, 2H); ^13^C NMR δ 30.5, 55.2, 69.1, 70.0, 72.6, 73.3, 76.1, 82.4, 114.0, 128.8, 129.7, 159.7; MS FAB *m*/*z* 336 (100, [M + Na]^+^); HRMS ESI *m*/*z* calcd for C_14_H_19_NO_7_Na [M + Na]^+^ 336.1059, found 336.1072.

**2-Chloro-3-*O*-(*p*-methoxybenzyl)-6-*O*-nitro-1,2,5-trideoxy-D-*arabino*-hexofuranose (11).** To a solution of Ph_3_P (2.18 g, 8.31 mmol) and DIAD (1.26 g, 1.22 mL, 6.23 mmol) in THF (14 mL) was added a solution of **10** (0.65 g, 2.08 mmol) in THF (6 mL) followed by freshly prepared HCl•pyridine (0.72 g, 6.23 mmol; prepared by slow addition of TMSCl (0.68 g, 0.8 mL, 6.31 mmol) to a solution of pyridine (0.98 g, 1.0 mL, 12.4 mmol) in MeOH (0.32 g, 0.4 mL, 9.9 mmol) and CH_2_Cl_2_ (10 mL) at 0 °C. Volatiles were evaporated and the crystalline residue was dried at 80 °C under vacuum overnight). The suspension was stirred overnight at room temperature. Volatiles were evaporated, and the residue was column chromatographed (hexanes/EtOAc, 10:1 → 6:1) to give **11** (0.43 g, 62%): ^1^H NMR δ 2.05–2.10 (m, 2H), 3.82 (br s, 4H), 3.81–3.86 (m, 1H), 3.90 (br d, *J* = 3.4 Hz, 1H), 4.02 (d, *J* = 10.3 Hz, 1H), 4.13 (dd, *J* = 4.4, 10.7 Hz, 1H), 4.47–4.65 (m, 4H), 6.90–6.93 (m, 2H), 7.25–7.28 (m, 2H); ^13^C NMR δ 30.9, 55.2, 60.1, 70.0, 72.1, 74.2, 80.9, 89.8, 113.9, 128.8, 129.6, 159.5; HRMS ESI *m*/*z* Calcd for C_14_H_18_ClNO_6_Na [M + Na]^+^ 354.0720, found 354.0706.

**2-Chloro-1,2,5-trideoxy-6-*O*-nitro-D-*arabino*-hexofuranose (13).** A solution of **11** (0.43 g, 1.30 mmol) and CAN (1.42 g, 2.59 mmol) in CH_3_CN (7 mL) and H_2_O (0.7 mL) was stirred for 1.5 h. The reaction mixture was concentrated, and the residue was deposited on silica gel and column chromatographed (hexanes/EtOAc, 10:1 → 3:1) to give **13** (0.21 g, 78%): ^1^H NMR δ 2.10–2.22 (m, 2H), 2.69 (br s, 1H), 3.80–3.84 (m, 1H), 4.02 (br d, *J* = 10.8 Hz, 1H), 4.14–4.25 (m, 3H), 4.58–4.68 (m, 2H); ^13^C NMR δ 30.9, 62.3, 70.0, 73.3, 81.7, 83.2; MS FAB *m*/*z* 212 (5, [M + H]^+^), 120 (100); HRMS ESI *m*/*z* calcd for C_6_H_11_ClNO_5_ [M + H]^+^ 212.0326, found 212.0327.

**2-Bromo-1,2,5-trideoxy-6-*O*-nitro-D-*arabino*-hexofuranose (14).** Step a: To a solution of Ph_3_P (2.85 g, 10.09 mmol) and DIAD (1.65 g, 8.15 mmol) in THF (30 mL) was added a stirred solution of **10** (1.75 g, 5.43 mmol) in THF (10 mL) followed by freshly prepared HBr•pyridine (1.30 g, 8.15 mmol; prepared by slow addition of TMSBr (0.93 g, 0.8 mL, 6.06 mmol) to a solution of pyridine (0.98 g, 1.0 mL, 12.4 mmol) in MeOH (0.32 g, 0.4 mL, 9.9 mmol) and CH_2_Cl_2_ (10 mL) at 0 °C. Volatiles were evaporated and the crystalline residue was dried at 80 °C under vacuum overnight). The suspension was stirred at room temperature for 22 h. Volatiles were evaporated, and the residue was column chromatographed (hexanes/EtOAc, 10:1 → 6:1) to give crude 2-bromo-3-*O*-(*p*-methoxybenzyl)-6-*O*-nitro-1,2,5-trideoxy-D-arabino-hexofuranose (**12**; 1.0 g, 49%) which was directly used in next step. Step *b*. A solution of crude **12** (1.0 g, 2.66 mmol) and CAN (2.91 g, 5.32 mmol) in a mixture of CH_3_CN (14 mL) and H_2_O (1.4 mL) was stirred for 1.5 h. The resulting mixture was concentrated and the residue was deposited on silica and column chromatographed (hexanes/EtOAc, 10:1 → 4:1) to give **14** (0.54 g, 79%): ^1^H NMR δ 2.14–2.26 (m, 2H), 3.80–3.86 (dt, *J* = 5.2, 8.8 Hz, 1H), 4.10 (dd, *J* = 4.0, 10.6 Hz, 1H), 4.16–4.22 (m, 1H), 4.26–4.33 (m, 2H), 4.58–4.72 (m, 2H); ^13^C NMR δ 31.2, 51.5, 69.9, 73.6, 81.5, 83.5. HRMS ESI/DART *m*/*z* calcd for C_6_H_14_^79^BrN_2_O_5_ [M + NH_4_]^+^ 273.0081, found 273.0084; calcd for C_6_H_14_^81^BrN_2_O_5_ [M + NH_4_]^+^ 275.0061, found 275.0061.

**1,5-Dideoxy-3-*O*-(*p*-methoxybenzyl)-6-*O*-nitro-*D-arabino*-hexofuranose (16b)**. Step a: A solution of compound **10** (2.70 g, 8.63 mmol) in THF (8 mL) followed by a solution of DIAD (2.09 g, 2.03 mL, 10.35 mmol) in THF (3 mL) were slowly (12 min) added to a stirred solution of Ph_3_P (2.71 g, 10.35 mmol) and PhCO_2_H (1.26 g, 10.35 mmol) in THF (40 mL) at –50 °C under nitrogen atmosphere. The resulting mixture was allowed to warm to ambient temperature within 1 h (it became colorless at –20 °C). Volatiles were evaporated, and the residue was column chromatographed (hexanes → hexanes/EtOAc, 3:1) to give 2-*O*-benzoyl-1,5-dideoxy-3-*O*-(*p*-methoxybenzyl)-6-*O*-nitro-*D-arabino*-hexofuranose (**16a**; 2.87 g, 80%): ^1^H NMR δ 2.0–2.07 (m, 2H), 3.79 (s, 3H), 3.85 (td, *J* = 1.0, 4.6 Hz, 1H), 3.90 (td, *J* = 5.0, 7.6 Hz, 1H), 4.08–4.12 (m, 2H), 4.51–4.59 (m, 3H), 4.75 (d, *J* = 11.7 Hz, 1H), 5.43 (td, *J* = 1.4, 2.7 Hz, 1H), 6.86 (d, *J* = 8.7 Hz, 2H), 7.27 (d, *J* = 8.7, 2H), 7.47 (t, *J* = 7.7 Hz, 2H), 7.61 (tt, *J* = 1.5, 7.5 Hz, 1H), 8.03 (d, *J* = 7.6 Hz, 2H). Step b: A suspension of KOH (0.40 g, 7.14 mol) in MeOH (20 mL) was added to a stirred solution of **16a** (2.82 g, 6.76 mmol) in MeOH (20 mL) at ambient temperature. After 1 h, the solution was neutralized with AcOH to pH 7. The volatiles were evaporated and the residue was column chromatographed (hexanes/EtOAc, 3:1 → 2:1) to give **16b** (1.64 g, 77%): ^1^H NMR δ 1.26 (d, *J* = 2.9 Hz, 1H), 2.00–2.04 (m, 2H), 3.64 (d, *J* = 2.9 Hz, 1H), 3.80 (br s, 4H), 3.83 (d, *J* = 10.3 Hz, 1H), 3.91 (dd, *J* = 3.9, 10.3 Hz, 1H), 4.27–4.29 (m, 1H), 4.48–4.58 (m, 4H), 6.88–6.91 (m, 2H), 7.24–7.27 (m, 2H); ^13^C NMR δ 30.9, 55.2, 70.2, 71.8, 74.1, 76.1 80.3, 89.2, 113.9, 128.4, 129.5, 159.4; MS FAB *m/z* 336 (100, [M + Na]^+^); HRMS ESI *m*/*z* calcd for C_14_H_19_NO_7_Na [M + Na]^+^ 336.1059, found 336.1047.

**1,5-Dideoxy-6-*O*-nitro-2-*O*-tosyl-D-*arabino*-hexofuranose (18)**. Step a: TsCl (134 mg, 0.70 mmol) was added to a stirred solution of **16b** (100 mg, 0.32 mmol) in anhydrous pyridine (1mL) at ambient temperature. After 16 h, the volatiles were evaporated and residue was partitioned between ice-cold AcOH/H_2_O (1:99, 30 mL) and CHCl_3_ (30 mL). The organic layer was separated, and the aqueous layer was extracted with CHCl_3_ (30 mL). Combined organic phase was washed with ice-cold saturated NaHCO_3_/H_2_O (30 mL), brine (30 mL) and dried (Na_2_SO_4_), concentrated in vacuo and column chromatographed (EtOAc/hexane, 5:95 → 35:65) to give 1,5-dideoxy-3-*O*-(*p*-methoxybenzyl)-6-*O*-nitro-2-*O*-tosyl-D-*arabino*-hexofuranose (**17a**; 128 mg, 86%) as a colorless oil: ^1^H NMR δ 1.90–1.96 (m, 2H), 2.46 (s, 3H), 3.72–3.78 (m, 1H), 3.82 (s, 3H), 3.84 (dt, *J* = 1.2, 4.6 Hz, 1H), 3.87 (d, *J* = 4.0 Hz, 1H), 3.92 (d, *J* = 11.3 Hz, 1H), 4.33 (d, *J* = 11.3 Hz, 1H), 4.42–4.49 (m, 3H), 4.93 (dt, *J* = 1.2, 4.0 Hz, 1H), 6.87 (d, *J* = 8.7 Hz, 2H), 7.16 (d, *J* = 8.7 Hz, 2H), 7.38 (d, *J* = 8.2 Hz, 2H), 7.81 (d, *J* = 8.2 Hz, 2H); ^13^C NMR δ 21.6, 30.3, 55.3, 69.8, 71.4, 71.8, 80.2, 83.9, 86.3, 114.0, 127.8, 128.8, 129.5, 130.1, 133.4, 145.4, 159.6. Step b: A solution of CAN (352 mg, 0.64 mmol) and **17a** (100 mg, 0.21 mmol) in MeCN (1 mL) and H_2_O (0.1 mL) was stirred at ambient temperature for 22 h. Volatiles were evaporated and the residue was column chromatographed (EtOAc/hexane, 3:97 → 30:70) to give **18** (73 mg, 98%) as a colorless oil: ^1^H NMR δ 1.97–2.07 (m, 1H), 2.07–2.17 (m, 1H), 2.47 (s, 3H), 3.69–3.76 (m, 1H), 3.85–3.90 (m, 1H), 3.95 (dd, *J* = 5.5, 11.3 Hz, 1H), 4.17 (dd, *J* = 2.5, 6.0 Hz, 1H), 4.51–4.63 (m, 2H), 4.76 (dt, *J* = 2.5, 5.5 Hz, 1H), 7.38 (d, *J* = 8.5 Hz, 2H), 7.80 (d, *J* = 8.5 Hz, 2H); ^13^C NMR δ 21.6, 30.2, 69.7, 70.3, 80.5, 80.9, 86.5, 127.9, 130.1, 132.8, 145.6. HRMS ESI/DART *m*/*z* calcd for C_13_H_21_N_2_O_8_S [M + NH_4_]^+^ 365.1013, found 365.1031.

**2-Chloro-1,2,5-trideoxy-3-*O*-(*p*-methoxybenzyl)-6-*O*-nitro-D-*ribo*-hexofuranose (19)**. Step a: TfCl (160 mg, 0.95 mmol) was added to a stirred solution of **16b** (250 mg, 0.80 mmol) and DMAP (295 mg, 2.4 mmol) in anhydrous CH_2_Cl_2_ (2 mL) at 0 °C (ice-bath). After 1 h, the reaction mixture was partitioned between ice-cold AcOH/H_2_O (1:99, 30 mL) and CH_2_Cl_2_ (30 mL). The aqueous layer was extracted with CH_2_Cl_2_ (30 mL) and the combined organic phase was washed with ice-cold saturated NaHCO_3_/H_2_O (30 mL), brine (30 mL) and dried (Na_2_SO_4_) to give 1,5-dideoxy-3-*O*-(*p*-methoxybenzyl)-2-*O*-(trifluoromethanesulfonyl)-6-*O*-nitro-D-*arabino*-hexofuraose as a colorless oil (**17b**; 313 mg, 88%) of sufficient purity to be used in next step. Column chromatography (EtOAc/hexane, 5:95 → 30:70) gave pure sample of **17b**: ^1^H NMR δ 1.93–2.02 (m, 2H), 3.80–3.84 (m, 1H), 3.82 (s, 3H), 3.92 (dt, *J =* 1.2, 4.6 Hz, 1H), 4.02 (dd, *J =* 3.5, 12.0 Hz, 1H), 4.18 (br d, *J =* 12.1 Hz, 1H), 4.47–4.52 (m, 3H), 4.66 (d, *J =* 11.5 Hz, 1H), 5.32 (d, *J =* 3.5 Hz, 1H), 6.91 (d, *J* = 8.8 Hz, 2H), 7.24 (d, *J* = 8.8 Hz, 2H). Step b: A solution of crude **17b** (50 mg, 0.11 mmol; from step a) and dried LiCl (23 mg, 0.54 mmol) in DMF (1 mL) was stirred for 5 h at ambient temperature under N_2_. Volatiles were evaporated and residue was partitioned between ice-cold saturated NaHCO_3_/H_2_O (15 mL) and EtOAc (15 mL). The separated organic phase was washed with brine (30 mL), dried (Na_2_SO_4_) and column chromatographed (EtOAc/hexane, 5:95 → 15:85) to give **19** (30 mg, 80.6%) as a colorless oil: ^1^H NMR δ 1.82–1.91 (m, 1H), 2.01–2.1 (m, 1H), 3.72 (dd, *J* = 5.2, 8.2 Hz, 1H), 3.82 (s, 3H), 3.99 (dt, *J* = 4.0, 8.2 Hz, 1H), 4.06 (dd, *J =* 2.8, 10.7 Hz, 1H), 4.30 (dd, *J* = 5.07,10.7 Hz, 1H), 4.40 (d, *J* = 11.2 Hz, 1H), 4.44 (dt, *J* = 2.2, 5.2 Hz, 2H), 4.48–4.59 (m, 2H), 4.69 (d, 11.3 Hz, 1H), 6.90 (d, *J* = 8.7 Hz, 2H), 7.30 (d, *J* = 8.7 Hz, 2H); ^13^C NMR δ 30.4, 55.2, 57.8, 69.9, 72.1, 74.0, 76.3, 81.5, 114.0, 128.8, 129.8, 159.7. HRMS ESI *m*/*z* calcd for C_14_H_18_^35^ClNO_6_Na [M + Na]^+^ 354.0715, found 354.0718; calcd for C_14_H_18_^37^ClNO_6_Na [M + Na]^+^ 356.0691, found 356.0694.

**2-Chloro-1,2,5-trideoxy-6-*O*-nitro-D-*ribo*-hexofuranose (20)**. A solution of CAN (74 mg, 0.13 mmol) and **19** (15 mg, 0.04 mmol) in MeCN (1mL) and H_2_O (0.1 mL) was stirred at ambient temperature for 22 h. Volatiles were evaporated and the residue was column chromatographed (EtOAc/hexane, 3:97 → 30:70) to give **20** (6.2 mg, 66%) as a colorless oil: ^1^H NMR δ 1.87–1.98 (m, 1H), 2.11–2.20 (m, 1H), 2.25 (d, *J* = 8.7 Hz, 1H), 3.82–3.88 (m, 1H), 3.93–4.00 (m, 1H), 4.02 (dd, *J* = 4.0, 10.7 Hz, 1H), 4.33 (dd, *J* = 5.4, 10.5 Hz, 1H), 4.44–4.48 (m, *J* = 4.2, 5.3 Hz, 1H), 4.55–4.67 (m, 2H); ^13^C NMR δ 30.5, 61.1, 69.8, 72.9, 75.7, 78.3; HRMS ESI/DART *m*/*z* calcd for C_6_H_14_^35^ClN_2_O_5_ [M + NH_4_]^+^ 229.0586, found 229.0588; calcd for C_6_H_14_^37^ClN_2_O_5_ [M + NH_4_]^+^ 231.0562, found 231.0561.

**2-Bromo-1,2,5-trideoxy-3-*O*-(*p*-methoxybenzyl)-6-*O*-nitro-D-*ribo*-hexofuranose (21)**. A solution of **17b** (30 mg, 0.06 mmol; prepared as described for **19**, step a) and dried LiBr (29 mg, 0.33 mmol) in DMF (1 mL) was stirred for 7 h at ambient temperature under N_2_. Volatiles were evaporated and the resulting residue was partitioned between ice-cold saturated NaHCO_3_/H_2_O (15 mL) and EtOAc (15 mL). The separated organic phase was washed with brine (30 mL), dried (Na_2_SO_4_) and column chromatographed (EtOAc/hexane, 5:95 → 15:85) to give **21** (18 mg, 71%) as a colorless oil: ^1^H NMR δ 1.83–1.94 (m, 1H), 2.02–2.12 (m, 1H), 3.6 (dd, *J* = 5.0, 7.6 Hz, 1H), 3.84 (s, 3H), 4.03 (dt, *J* = 4.0, 8.0 Hz, 1H), 4.22 (dd, *J* = 2.8, 10.6 Hz, 1H), 4.44 (d, *J* = 11.1 Hz, 1H), 4.43–4.51 (m, 2 H), 4.51–4.61 (m, 2H), 4.71 (d, *J* = 11.1 Hz, 1H) 6.92 (d, *J* = 8.6 Hz, 2H), 7.33 (d, *J* = 8.6 Hz, 2H); ^13^C NMR δ 30.4, 49.7, 55.3, 69.9, 72.1, 74.1, 76.6, 81.2, 114.0, 128.81, 129.8, 159.71. HRMS ESI *m*/*z* calcd for C_14_H_18_^79^BrNO_6_Na [M + Na]^+^ 398.0210, found 398.0203; calcd for C_14_H_18_^81^BrNO_6_Na [M + Na]^+^ 400.0191, found 400.0183.

**2-Bromo-1,2,5-trideoxy-6-*O*-nitro-D-*ribo*-hexofuranose (22)**. A solution of CAN (78 mg, 0.14 mmol) and **21** (18 mg, 0.05 mmol) in MeCN (1 mL) and water (0.1 mL) was stirred at ambient temperature for 22 h. Volatiles were evaporated and the residue was column chromatographed (EtOAc/hexane, 3:97 → 30:70) to give **22** (12 mg, 96%) as a colorless oil: ^1^H NMR δ 1.89–1.98 (m, 1H), 2.11–2.20 (m, 2H), 3.78–3.84 (m, 1H), 3.85–3.92 (m, 1H), 4.13 (dd, *J* = 4.6 Hz, 1H), 4.43 (dd, *J* = 5.4 Hz, 1H), 4.48 (dd, *J* = 5.0 Hz, 1H), 4.55–4.70 (m, 2H); ^13^C NMR δ 31.6, 53.7, 69.8, 72.9, 75.3, 78.8. HRMS ESI/DART *m/z* calcd for C_6_H_14_^79^BrN_2_O_5_ [M + NH_4_]^+^ 273.0081, found 273.0077; calcd for C_6_H_14_^81^BrN_2_O_5_ [M + NH_4_]^+^ 275.0066, found 275.0058.

**1,5-Dideoxy-3-*O*-methyl-6-*O*-nitro-D-*ribo*-hexofuranose (23)** and **1,5-Dideoxy-2-*O*-methyl- 6-*O*-nitro-D-*ribo*-hexofuranose (24)** and: A suspension of **8** (0.34 g, 1.76 mmol) and Bu_2_SnO (0.44 g, 1.76 mmol) in anhydrous MeOH (8 mL) was refluxed for 30 min. Volatiles were evaporated after the flask was cooled to ambient temperature. DMF (1 mL) and MeI (1.14 g, 0.5 mL, 8.03 mmol) were added, the flask was sealed and the solution was stirred at 40 °C for 12 h. Volatiles were evaporated and the residue was column chromatographed (hexanes/EtOAc, 4:1 → 1:1) to give **23** (145 mg, 38%) and **24** (134 mg, 35%). Compound **23** had: ^1^H NMR δ 1.85–1.92 (m, 1H), 2.09–2.16 (m, 1H), 2.79 (d, *J* = 8.8 Hz, 1H), 3.44 (s, 3H), 3.67 (ddd, *J* = 3.9, 7.8, 8.8 Hz, 1H), 3.74–3.84 (m, 3H), 4.06 (dd, *J* = 4.4, 5.4 Hz, 1H), 4.56–4.65 (m, 2H); ^13^C NMR δ 30.5, 57.7, 70.0, 70.1, 75.4, 78.6, 79.4; MS FAB *m/z* 230 (100, [M + Na]^+^); HRMS ESI *m*/*z* calcd for C_7_H_13_NO_6_Na [M + Na]^+^ 230.0641, found 230.0651. Compound **24** had: ^1^H NMR δ 1.89–1.96 (m, 1H), 2.04–2.11 (m, 1H), 2.70 (d, *J* = 3.9 Hz, 1H), 3.45 (dd, *J* = 4.9, 6.8 Hz, 1H), 3.47 (s, 3H), 3.79 (dd, *J* = 2.9, 9.8 Hz, 1H), 3.86 (ddd, *J* = 3.9, 6.8, 8.3 Hz, 1H), 3.79 (dd, *J* = 4.4, 10.2 Hz, 1H), 4.31 (m, 1H), 4.55–4.65 (m, 2H); ^13^C NMR δ 30.8, 58.3, 68.9, 70.0, 73.2, 75.9, 85.2; MS FAB *m/z* 208 (100, [M + H]^+^); HRMS ESI *m*/*z* calcd for C_7_H_14_NO_6_ [M + H]^+^ 208.0821, found 208.0819.

**1,5-Dideoxy-2-*O*-tosyl-3-*O*-methyl-6-*O*-nitro-D-*ribo*-hexofuranose (25).** TsCl (101 mg, 0.53 mmol) was added to a stirred solution of **23** (100 mg, 0.48 mmol) in anhydrous pyridine (1 mL) at ambient temperature. After 16 h, the volatiles were evaporated and residue was partitioned between ice-cold AcOH/H_2_O (1:99, 30 mL) and CHCl_3_ (30 mL). The organic layer was separated, and the aqueous layer was extracted with CHCl_3_ (30 mL). Combined organic phase was washed with ice-cold NaHCO_3_/H_2_O (30 mL), brine (30 mL), dried (Na_2_SO_4_), concentrated in vacuo and column chromatographed (EtOAc/hexane, 5:95 → 35:65) to give **25** (110 mg, 63%) as a colorless oil: ^1^H NMR δ 1.86–1.96 (m, 1H), 2.05–2.15 (m, 1H), 2.48 (s, 3H), 3.31 (s, 3H), 3.47 (dd, *J* = 5.0, 8.6 Hz, 1H), 3.85 (dd, *J* = 4.2, 8.6 Hz, 1H), 3.89 (dd, *J* = 2.44, 11.5 Hz, 1H), 4.07 (dd, *J* = 4.44, 11.5 Hz, 1H), 4.51–4.63 (m, 2H), 5.12 (dt, *J* = 2.2, 4.7 Hz, 1H), 7.39 (d, *J* = 8.15 Hz, 2H), 7.85 (d, *J =* 8.15 Hz, 2H); ^13^C NMR δ 21.6, 30.5, 58.3, 69.8, 70.9, 75.9, 76.7, 83.7, 127.8, 129.9, 133.7, 145.2; HRMS ESI/DART *m*/*z* calcd for C_14_H_23_N_2_O_8_S [M + NH_4_]^+^ 379.1170, found 379.1172.

**2-*O*-Benzoyl-1,5-dideoxy-3-*O*-methyl-6-*O*-nitro-D-*arabino*-hexofuranose (26).** Compound **23** (0.20 g, 0.97 mmol) in THF (6 mL) was added to a stirred solution of Ph_3_P (0.31 g, 1.16 mmol) and PhCO_2_H (0.14 g, 1.16 mmol) in THF (5 mL) at −50 °C. After 5 min. DIAD (0.23 g, 0.22 mL, 1.16 mmol) in THF (2 mL) was added slowly over 12 min. The reaction mixture was allowed to warm to room temperature within 1 h (it became colorless at −20 °C). Volatiles were evaporated, and the residue was column chromatographed (hexanes → hexanes/EtOAc, 10:1) to give **26** (0.23 g, 76%): ^1^H NMR δ 2.07–2.17 (m, 2H), 3.50 (s, 3H), 3.71 (d, *J* = 3.4 Hz, 1H), 3.86–3.90 (m, 1H), 4.03–4.07 (m, 2H), 4.57–4.66 (m, 2H), 5.38–5.40 (m, 1H), 7.44–7.62 (m, 3H), 8.02–8.14 (m, 2H); ^13^C NMR δ 30.8, 58.0, 70.0, 71.7, 77.9, 80.4, 89.6, 128.4, 129.4, 130.1, 133.4, 165.7; MS FAB *m*/*z* 334 (15, [M + Na]^+^), 312 (100, [M + H]^+^); HRMS ESI *m*/*z* calcd for C_14_H_17_O_7_NNa [M + Na]^+^ 334.0903, found 334.0904.

**1,5-Dideoxy-3-*O*-methyl-6-*O*-nitro-D-*arabino*-hexofuranose (27).** KOH (1.62 g, 28.9 mmol) in MeOH (80 mL) was added to a stirred solution of **26** (1.82 g, 5.85 mmol) in MeOH (80 mL). The reaction mixture was left to stir at room temperature for 1 h, then was neutralized with 5% HCl/H_2_O. Volatiles were evaporated, and the residue was column chromatographed (hexanes/EtOAc, 3:1 → 1:1) to give **27** (0.87 g, 72%): ^1^H NMR (300 MHz) δ 2.07–2.14 (m, 2H), 2.80 (br s, 1H), 3.42 (s, 3H), 3.48 (td, *J* = 1.1, 3.9 Hz, 1H), 3.79 (ddd, *J* = 3.9, 6.1, 7.3 Hz, 1H), 3.85 (br d, *J* = 10.3 Hz, 1H), 3.90 (dd, *J* = 3.7, 10.3 Hz, 1H), 4.27–4.29 (m, 1H), 4.54–4.67 (m, 2H); ^13^C NMR δ 30.9, 57.5, 70.2, 73.9, 75.2, 80.2, 91.6; MS FAB *m*/*z* 208 (5, [M + H]^+^), 71 (100); HRMS ESI *m*/*z* calcd for C_7_H_14_NO_6_ [M + H]^+^ 208.0821, found 208.0807.

**2-Chloro-3-*O*-methyl-6-*O*-nitro-1,2,5-trideoxy-D-*arabino*-hexofuranose (28).** Solution of **23** (0.60 g, 2.90 mmol) in THF (15 mL) was added to a stirred solution of Ph_3_P (1.52 g, 5.78 mmol) and DIAD (0.89 g, 0.86 mL, 4.38 mmol) in THF (15 mL) followed by addition of freshly prepared HCl•pyridine (0.50 g, 4.33 mmol). The suspension was stirred overnight at room temperature. Volatiles were evaporated, and the residue was chromatographed (hexanes → hexanes/EtOAc, 6:1) to give **28** (0.38 g, 61%): ^1^H NMR δ 2.14–2.18 (m, 2H), 3.44 (s, 3H), 3.73 (d, *J* = 3.9 Hz, 1H), 3.78–3.82 (m, 1H), 4.02 (d, *J* = 10.7 Hz, 1H), 4.10 (dd, *J* = 4.4, 10.7 Hz, 1H), 4.24–4.27 (m, 1H), 4.56–4.67 (m, 2H); ^13^C NMR δ 31.3, 58.1, 59.4, 70.0, 74.2, 80.9, 92.8; HRMS ESI/DART *m*/*z* calcd for C_7_H_16_^35^ClN_2_O_5_ [M + NH_4_]^+^ 243.0742, found 243.0752; calcd for C_7_H_16_^37^ClN_2_O_5_ [M + NH_4_]^+^ 245.0716, found 245.0715.

**2-Bromo-3-*O*-methyl-6-*O*-nitro-1,2,5-trideoxy-D-*arabino*-hexofuranose (29).** Solution of **23** (0.65 g, 3.13 mmol) in THF (15 mL) was added to a stirred solution of Ph_3_P (1.64 g, 6.25 mmol) and DIAD (0.95 g, 0.92 mL, 4.69 mmol) in THF (15 mL) followed by addition of freshly prepared HBr•pyridine (0.75 g, 4.69 mmol). The suspension was stirred overnight at room temperature. Volatiles were evaporated, and the residue was column chromatographed (hexanes/EtOAc, 10:1 → 6:1) to give **29** (0.39 g, 46%): ^1^H NMR δ 2.19–2.27 (m, 2H), 3.49 (s, 3H), 3.82 (dt, *J* = 4.2, 6.8 Hz, 1H), 3.92 (d, *J* = 3.8 Hz, 1H), 4.14 (dd, *J* = 1.86, 11.0 Hz, 1H), 4.21 (dd, *J* = 4.47, 11.0 Hz, 1H), 4.27–4.31 (m, 1H), 4.58–4.70 (m, 2H); ^13^C NMR δ 31.5, 48.5, 58.0, 70.0, 74.6, 81.2, 93.0; HRMS ESI/DART *m*/*z* calcd for C_7_H_16_^79^BrN_2_O_5_ [M + NH_4_]^+^ 287.0237, found 287.0252; calcd for C_7_H_16_^81^BrN_2_O_5_ [M + NH_4_]^+^ 289.0218, found 289.0233.

**1,5-Dideoxy-2-*O*-tosyl-3-*O*-methyl-6-*O*-nitro-D-*arabino*-hexofuranose (30).** TsCl (116 mg, 1.057 mmol) was added to a stirred solution of **27** (200 mg, 0.965 mmol) in anhydrous pyridine (1 mL) at ambient temperature. After 16 h, the volatiles were evaporated and residue was partitioned between ice-cold AcOH/H_2_O (1:99, 30 mL) and CHCl_3_ (30 mL). The organic layer was separated, and the aqueous layer was extracted with CHCl_3_ (30 mL). Combined organic phase was washed with ice-cold saturated NaHCO_3_/H_2_O (30 mL), brine (30 mL) and dried (Na_2_SO_4_), concentrated in vacuo and column chromatographed (EtOAc/hexane, 5:95 → 35:65) to give **30** (240 mg, 69%) as a colorless oil: ^1^H NMR δ 1.96–2.11 (m, 2H), 2.47 (s, 3H), 3.29 (s, 3H), 3.65 (td, *J* = 1.2, 4.4 Hz, 1H), 3.69–3.75 (m, 1H), 3.81 (dd, *J* = 4.1, 11.4 Hz, 1H), 3.92 (d, *J* = 11.4 Hz, 1H), 4.48–4.60 (m, 2H), 4.86 (td, *J* = 1.2, 4.1 Hz, 1H), 7.38 (d, *J* = 8.4 Hz, 2H), 7.81 (d, *J* = 8.4 Hz, 2H). ^13^C NMR δ 21.6, 30.5, 58.0, 69.8, 71.3, 80.1, 83.1, 89.4, 127.8, 130.0, 133.4, 145.4; HRMS ESI/DART *m*/*z* calcd for C_14_H_23_N_2_O_8_S [M + NH_4_]^+^ 379.1170, found 379.1169.

**2-Chloro-1,2,5-trideoxy-3-*O*-methyl-6-*O*-nitro-D-*ribo*-hexofuranose (32).** Step a: TfCl (123 μL, 195 mg, 1.16 mmol) was added to a stirred solution of **27** (200 mg, 0.96 mmol) and DMAP (354 mg, 2.9 mmol) in anhydrous CH_2_Cl_2_ (2 mL) at 0 °C (ice-bath). After 1 h, the reaction mixture was partitioned between ice-cold AcOH/H_2_O (1:99, 30 mL) and CH_2_Cl_2_ (30 mL). The aqueous layer was extracted with CH_2_Cl_2_ (30 mL) and the combined organic phase was washed with ice-cold saturated NaHCO_3_/H_2_O (30 mL), brine (30 mL) and dried (Na_2_SO_4_) to give **31** as a colorless oil (243 mg, 74%) of sufficient purity to be used in next step. Column chromatography (EtOAc/hexane, 5:95 → 30:70) gave pure sample of **31**: ^1^H NMR δ 2.01–2.09 (m, 1H), 2.09–2.19 (m, 1H), 3.46 (s, 3H), 3.75–3.82 (m, 2H), 3.98 (dd, *J =* 3.6, 12.1 Hz, 1H), 4.17 (d, *J =* 12.1 Hz, 1H), 4.53–4.65 (m, 2H), 5.26 (d, *J =* 3.5 Hz, 1H); *Step b*. A solution of crude **31** (100 mg, 0.29 mmol; from step a) and dried LiCl (62.5 mg, 1.47 mmol) in DMF (1 mL) was stirred for 5 h at ambient temperature under N_2_. Volatiles were evaporated and residue was partitioned between ice-cold saturated NaHCO_3_/H_2_O (15 mL) and EtOAc (15 mL). The separated organic phase was washed with brine (15 mL), dried (Na_2_SO_4_) and column chromatographed (EtOAc/hexane, 5:95 → 15:85) to give **32** (49 mg, 74%) as a colorless oil: ^1^H NMR δ 1.89–2.02 (m, 1H), 2.06–2.18 (m, 1H), 3.44 (s, 3H), 3.58 (dd, *J =* 5.1, 8.0 Hz, 1H), 3.95 (td, *J* = 4.1, 8.3 Hz, 1H), 4.05 (dd, *J =* 2.7, 10.8 Hz, 1H), 4.32 (dd, *J =* 4.8, 10.8 Hz, 1H), 4.49 (td, *J* = 2.8, 5.0 Hz, 1H), 4.53–4.64 (m, 2H); ^13^C NMR δ 30.7, 57.6, 58.1, 69.9, 73.9, 76.1, 84.7; HRMS ESI/DART *m*/*z* calcd for C_7_H_16_^35^ClN_2_O_5_ [M + NH_4_]^+^ 243.0742, found 243.0747; calcd for C_7_H_16_^37^ClN_2_O_5_ [M + NH_4_]^+^ 245.0718, found 245.0716.

**2-Bromo-1,2,5-trideoxy-3-*O*-methyl-6-*O*-nitro-D-*ribo*-hexofuranose (33).** A solution of crude **31** (100 mg, 0.29 mmol; prepared as described for **32**, step a) and dried LiBr (77 mg, 0.88 mmol) in DMF (1 mL) was stirred for 5 h at ambient temperature under N_2_. Volatiles were evaporated and residue was partitioned between ice-cold saturated NaHCO_3_/H_2_O (15 mL) and EtOAc (15 mL). The separated organic phase was washed with brine (15 mL), dried (Na_2_SO_4_) and column chromatographed (EtOAc/hexane, 5:95 → 15:85) to give **33** (41 mg, 52%) as a colorless oil: ^1^H NMR δ 1.90–2.0 (m, 1H), 2.06–2.16 (m, 1H), 3.39–3.45 (m, 4H), 3.97 (td, *J* = 4.2, 8.0 Hz, 1H), 4.20 (dd, *J* = 3.0, 11 Hz, 1H), 4.45 (dd, *J =* 4.8, 11.0 Hz, 1H), 4.50 (td, *J* = 2.9, 5.0 Hz, 1H), 4.53–4.64 (m, 2H); ^13^C NMR δ 30.7, 49.5, 58.2, 69.9, 74.0, 76.5, 84.4; HRMS ESI/DART *m*/*z* calcd for C_7_H_16_^79^BrN_2_O_5_ [M + NH_4_]^+^ 287.0237, found 287.0239; calcd for C_7_H_16_^81^BrN_2_O_5_ [M + NH_4_]^+^ 289.0222, found 289.0218.

**Biomimetic studies with 6-*O*-nitro-1,4-anhydrohexitols. Typical Procedure**. A solution of **9** (20 mg, 0.06 mmol), Bu_3_SnH (77 μL, 83 mg, 0.28 mmol), and AIBN (18 mg, 0.12 mmol) in dried toluene (2 mL) was deoxygenated (Ar) for 20 min and then heated at 95 °C for 1 h. Volatiles were evaporated carefully (at 25 °C and diminished pressure ~40 mmHg) and the residue was purified by column chromatography (EtOAc/hexane, 10:90 → 70:30) to give 1,2,5-trideoxy-D-*glycero*-hexofuranose-3-ulose **34a** in equilibrium mixture (~1:1) with cyclic hemiacetal **35a** (5 mg, 67%) as a colorless oil: HRMS ESI/DART *m*/*z* calcd for C_6_H_11_O_3_ [M + H]^+^ 131.0703, found 131.0707. Ketone **34a** had: ^1^H NMR δ 1.84–1.93 (m, 1H), 1.95–2.06 (m, 2H), 2.15–2.27 (m, 2H), 3.74–3.85 (m, 2H), 3.88 (dd, *J* = 4.9, 7.4 Hz, 1H), 4.04–4.16 (m, 2H); ^13^C NMR δ 32.9, 38.9, 59.8, 68.9, 78.6, 215.9. Hemiacetal **35a** had: 1.95–2.06 (m, 1H), 2.15–2.27 (m, 1H), 2.46–2.64 (m, 3H), 3.93–4.03 (m, 2H), 4.04–4.16 (m, 1H) 4.28 (dd, *J* = 2.27, 5.6 Hz, 1H), 4.37 (dt, *J* = 4.1, 9.2 Hz, 1H); ^13^C NMR δ 32.1, 36.7, 64.6, 68.3, 85.7, 114.9.

Also isolated from the reaction mixture was 1,5-dideoxy-2-*O*-tosyl-D-*ribo*-hexofuranose (3.3 mg, 19%): ^1^H NMR δ 1.81–1.94 (m, 2H), 2.46 (s, 3H), 3.74–3.85 (m, 4H), 3.86–3.93 (m, 1H), 4.12 (dd, *J* = 4.9, 11.2 Hz, 1H), 4.95 (dt, *J* = 3.0, 5.3 Hz, 1H), 7.38 & 7.82 (2 × d, *J* = 8.3 Hz, 2 × 2H).

Analogous treatment of **9** (20 mg, 0.06 mmol) with Bu_3_SnD (77 μL, 83 mg, 0.28 mmol), instead of Bu_3_SnH gave 2-deuterio epimers (2*R/S*, ~1:1) of **34b** in equilibrium mixture (~1:1) with **35b** (5.2 mg, 71%) as a colorless oil: ^1^H NMR spectrum of **34b**/**35b** corresponded to this of the above **34a**/**35a** with reduction of the integrated intensity for the H2/2′ signal at δ 2.15–2.27 and 2.46–2.64 to approximately half and simplification of the H1/1′ signals at δ 4.04–4.16 (m, 1H) and 4.33–4.40 (m, 1H). ^13^C NMR spectrum of **34b**/**35b** showed triplets at δ 36.7 and 38.9 (*J* = 20.1 Hz) for C2 carbons because of splitting to deuterium and two close peaks of equal intensity for each hemiacetal carbons. HRMS ESI/DART *m*/*z* calcd for C_6_H_10_DO_3_ [M + H]^+^ 132.0765, found 132.0768. Isotopic incorporation (MS) was calculated to be 85–95% for [^2^H] isotopomers of **34b/35b** depends on the experiments. The ^13^C NMR spectrum for the sample of **34b**/**35b** (2*R/S*, ~1:1, [^2^H] incorporation ~85%) showed residual peaks at 38.9 ppm and 36.7 for the unlabeled **34a** and **35a**, respectively and isotopically upfield shifted carbon signals for **34b** (two sets of triplets of equal intensity at 38.60 and 38.63 ppm with *J*_C2–D_ = 20.1 Hz) and **35b** two sets of triplets of equal intensity at 36.35 and 3.36 with *J*_C2–D_ = 20.1 Hz), respectively.

**6-*O*-Benzoyl-1,2,5-trideoxy-D-*glycero*-hexofuranose-3-ulose** (**36a)**. BzCl (23 µL, 28 mg, 0.2 mmol), pyridine (44 µL, 43 mg, 0.54 mmol), and DMAP (4 mg, 0.032 mmol) were added to a stirred solution of **34a**/**35a** (30 mg, 0.23 mmol), in CH_2_Cl_2_ (2 mL). Stirring was continued at ambient temperature for 3 h and MeOH (0.3 mL) was added. Volatiles were evaporated and the residue was chromatographed (EtOAc/hexane, 5:95 → 15:85) to give **36a** (22 mg, 81%) as an colorless oil: ^1^H NMR δ 2.06–2.16 (m, 1H), 2.18–2.28 (m, 1H), 2.51 (dd, *J* = 6.50, 8.26 Hz, 2H), 3.90 (dd, *J* = 4.71, 7.0 Hz, 1H), 4.05–4.13 (m, 1H), 4.28–4.36 (m, 1H), 4.39–4.46 (m, 1H), 4.47–4.54 (m, 1H), 7.41–7.47(m, 2H), 7.56 (tt, *J* = 1.5, 7.4 Hz, 1H), 8.0 (d, *J* = 8.57 Hz, 2H); ^13^C NMR δ 29.9, 36.8, 60.9, 64.7, 76.8, 128.5, 129.7, 130.2, 133.1, 166.5, 215.5; HRMS TOF/DART *m*/*z* calcd for C_13_H_18_NO_4_ [M + NH_4_]^+^ 252.1230, found 252.1234.

**6-*O*-Benzoyl-2-deuterio-1,2,5-trideoxy-D-*glycero*-hexofuranose-3-ulose** (**36b).** Treatment of **34b**/**35b** (30 mg, 0.23 mmol) with BzCl, as described for **36a**, gave **36b** (12 mg, 67%) as a colorless oil: ^1^H NMR spectrum of **36b** corresponded to this of the above **36a** with reduction of the integrated intensity for the H2/2′ signal at δ 2.51 to half and simplification of the H1/1′ signals at 4.05–4.13 and 4.28–4.36. ^13^C NMR spectrum showed triplet at δ 36.4 (*J* = 20.1 Hz) for C2 because of splitting to deuterium. HRMS ESI/DART *m*/*z* calcd for C_13_H_13_DNaO_4_ [M + Na]^+^ 258.0847, found 258.0836.

**Biomimetic studies with 3-*O*-methyl-6-*O*-nitro-1,4-anhydrohexitols. 2-(2-Hydroxyethyl)-3-methoxyfuran (37**). A solution of **25** (25 mg, 0.069 mmol), Bu_3_SnH (92 μL, 100 mg, 0.34 mmol), and AIBN (22.7 mg, 0.14 mmol) in dried toluene (2 mL), was deoxygenated (Ar) for 20 min and then heated at 95 °C for 1.5 h. Volatiles were evaporated and the residue was purified by column chromatography (EtOAc/hexane, 10:90 → 75:25) to give **37** (6.2 mg, 63%) followed by 1,5-dideoxy-2-*O*-tosyl-3-*O*-methyl-D-*ribo*-hexofuranose (**38**, 4.8 mg, 22%) as a colorless oils. Compound **37** had: ^1^H NMR δ 2.89 (t, *J* = 6.0 Hz, 2H), 3.76 (s, 3H), 3.86 (t, *J* = 6.0 Hz, 2H), 6.31 (d, *J* = 2.1 Hz, 1H), 7.16 (d, *J* = 2.1 Hz, 1H); ^13^C NMR δ 29.3, 59.4, 60.9, 102.9, 136.7, 139.7, 144.3; HRMS ESI/FT-ICR *m*/*z* calcd for C_7_H_11_O_3_ [M + H]^+^ 143.0702, found 143.0701. Compound **38** had: ^1^H NMR δ 1.74–1.84 (m, 1H), 1.85–1.94 (m, 1H), 2.29 (t, *J* = 5.9 Hz, 1H), 2.46 (s, 3H), 3.32 (s, 3H), 3.49 (dd, *J* = 4.9, 8.3 Hz, 1H), 3.72–3.79 (m, 2H), 3.85–3.94 (m, 2H), 4.07 (dd, *J* = 4.7, 11.3 Hz, 1H), 5.11 (dt, *J* = 2.4, 4.8 Hz, 1H), 7.36 (d, *J* = 8.1 Hz, 2H), 7.83 (d, *J* = 8.1 Hz, 2H); ^13^C NMR δ 21.6, 35.6, 58.3, 60.6, 70.9, 76.5, 79.1, 83.8, 127.8, 129.9, 133.8, 145.1; HRMS ESI/DART *m*/*z* calcd for C_14_H_21_O_6_S [M + H]^+^ 317.1053, found 317.1055.

Analogous treatment of **25** with Bu_3_SnD gave **37** (6.0 mg, 61%) and (**38**, 4.8 mg, 22%) with spectroscopic data as above.

Treatment of **30** (25 mg, 0.069 mmol) with Bu_3_SnH, as described for **37**, gave **37** (5.0 mg, 51%) with data as above and 1,5-dideoxy-2-*O*-tosyl-3-*O*-methyl-D-*arabino*-hexofuranose **39** (8.3 mg, 38%) as a colorless oil: ^1^H NMR δ 1.86–1.94 (m, 2H), 2.04–2.11 (m, 1H), 2.47 (s, 3H), 3.31 (s, 3H), 3.70 (dt, *J* = 1.2, 5.0 Hz, 1H), 3.73–3.82 (m, 3H), 3.83 (d, *J* = 4.3 Hz, 1H), 3.93 (d, *J* = 11.7 Hz, 1H), 4.88 (dt, *J* = 1.3, 4.2 Hz, 1H), 7.37 (d, *J =* 8.4 Hz, 2H), 7.81 (d, *J =* 8.4 Hz, 2H); ^13^C NMR δ 21.6, 35.3, 58.0, 60.5, 71.3, 82.7, 83.4, 89.7, 127.8, 130.0, 133.5, 145.3; HRMS ESI/DART *m*/*z* calcd for C_14_H_21_O_6_S [M + H]^+^ 317.1053, found 317.1050.

Analogous treatment of **30** with Bu_3_SnD gave **37** (4.9 mg, 50%) and **39** (8.2 mg, 38%) with spectroscopic data as above.

**1,2,5-Trideoxy-3-*O*-methyl-D-*glycero*-hex-2-enofuranose (40). Typical Procedure.** A solution of **29** (20 mg, 0.074 mmol), Bu_3_SnD (99 μL, 108 mg, 0.369 mmol), and AIBN (24 mg, 0.146 mmol) in dried toluene (2 mL), was deoxygenated (Ar) for 20 min and then heated at 95 °C for 2 h. Volatiles were evaporated and the residue was purified by column chromatography (EtOAc/hexane, 10:90 → 70:30) to give **40** [22] (5.0 mg, 47%) as a colorless oil: ^1^H NMR δ 1.73–1.87 (m, 1H), 1.94–2.09 (m, 1H), 2.58 (t, *J* = 5.6 Hz, 1H), 3.68 (s, 3H), 3.76–3.82 (m, 2H), 4.60–4.70 (m, 3H), 4.73–4.79 (m, 1H); ^13^C NMR δ 35.6, 57.6, 60.6, 72.8, 81.2, 90.2, 157.7; HRMS ESI/DART *m*/*z* calcd for C_7_H_16_NO_3_ [M + NH_4_]^+^ 162.1125, found 162.1131.

**Comparison of Reaction Rates of 9, 13 or 18 with Bu_3_SnH.** Independent solutions of 0.057 mmol samples of **9**, **13** and **18** in toluene–*d*_8_ (2.0 mL) were treated with 5 molar equiv of Bu_3_SnH and 2 molar equiv of AIBN at 75 °C. Aliquots of the individual reaction mixtures (0.3 mL) were diluted in toluene–*d*_8_ (0.2 mL) and directly analyzed by ^1^H NMR. The **34a/35a** (1:1)**/**starting material ratios were obtained by integrating disappearance of the peak at 4.55 ppm for H2 of **9** or **18** or at 3.96 ppm for H6 of **13** and appearance of the peak at 4.10 ppm for the H4 of **34a**. The determinations were conducted under the pseudo-first-order conditions:

*k*_1_t = −2.303 log(*C/C*_o_) + *a*

where *C/C*_o_ is the ratio of the concentration of starting material **9**, **18**, or **13** in the mixture at time *t* to the initial concentration of starting material. Values of the term [−log(*C/C*_o_)] were plotted against [*t*(min)*k*(s^−1^) = *k*_1_ (min^−1)^/3600].

## 5. Summary and Conclusions

Remarkable changes between anionic and radical elimination mechanisms occurred with our 1,4-anhydrofuranitols. All 2-(bromo, chloro, and tosylate) epimers that contained a 3-hydroxyl group underwent two-electron elimination of bromide, chloride, or tosylate ions to give the same furanone **34a** or deuterated furanone **34b**. The inductively donating C1 of the furanitols allows for the elimination of an anion from C2 upon generation of a radical center at C3. Loss of the 3-hydroxyl proton produces a 3-oxo-C2 radical, which abstracts hydrogen from HSnBu_3_ or deuterium from DSnBu_3_.

All of the 3-*O*-methyl-2-bromo- and 2-chloro analogues underwent one-electron elimination of a bromine or chlorine atom to produce 2-(2-hydroxyethyl)-3-methoxy-2,5-dihydrofuran **40**. No deuterium incorporation occurred in these cases. The 3-*O*-methyl-2-*O*-tosyl epimers underwent more drastic fragmentation upon generation of a C3 radical. Elimination of toluenesulfonic acid from C2/C1 and abstraction of hydrogen from C4 produced the aromatized 2-(2-hydroxyethyl)-3-methoxyfuran **37**.

The inductively negative anomeric carbon (C1′) in nucleos(t)ides disfavors elimination of a chloride anion from C2′. Loss of Cl• followed by 1,4-elimination of the 3′-hydroxyl proton and base gives the unlabeled 2-(2-hydroxyethyl)-3(2*H*)-furanone (path b, Figure 4). The abstraction of deuterium from DSnBu_3_ by Cl• provides a chain propagation step and so no label is incorporated into the furanone product.

By contrast, dissociation of a prohibitively high-energy tosyloxy radical is precluded so that departure of a tosylate anion from C2′ occurs. Loss of the 3′-hydroxyl proton gives a 3′-oxo-C2′ radical, which abstracts deuterium from DSnBu_3_ and undergoes elimination of H/D and nucleobase to produce 4-deuterio-2-(2-hydroxyethyl)-3(2*H*)-furanone (path a, Figure 4).

Our combined results with nucleoside and anhydroalditol models provide data for plausible mechanistic rationalization of the two-electron elimination of hydrogen-bonded water from substrate nucleoside di(or tri)phosphates, and for one-electron dissociation of a chlorine atom from 2′-chloro-2′-deoxynucleoside di(or tri)phosphate inactivators of ribonucleotide reductases and the MoaA enzyme. Such Cl• radicals could react with active site components and contribute to enzyme inactivation.

Theoretical studies [18,19,44] employ major assumptions and calculation simplifications relative to actual biological systems. Mechanistic hypotheses based on reactions executed by modified enzymes [26,46] also involve unnatural “biomimetic” models—whether prepared by chemical synthesis or molecular biology. Combination of theoretical, biochemical, and biomimetic modeling provides more insightful approximations that any one of the individual models. Results of our present studies add clarity to hypotheses postulated for the radical chemistry-based inactivation of RNRs [30] and MoaA [32].

## Figures and Tables

**Table 1:**
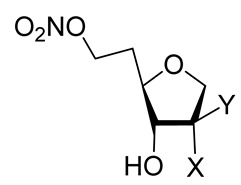
Products from 6-*O*-nitro-1,4-anhydrohexitols *^a.^*

Entry	Substrate			Reagent	Products	Ratio *^b^*	Yield *^c^* (%)
	No	X	Y				
1	**9**	OTs	H	Bu_3_SnH	**34a:35a**	1:1	66 *^d^*
2	**9**	OTs	H	Bu_3_SnD	**34b:35b**	1:1	71 *^d^*
3	**20**	Cl	H	Bu_3_SnH	**34a:35a**	1:1	81
4	**20**	Cl	H	Bu_3_SnD	**34b:35b**	1:1	67
5	**22**	Br	H	Bu_3_SnH	**34a:35a**	1:1	64
6	**22**	Br	H	Bu_3_SnD	**34b:35b**	1:1	68
7	**13**	H	Cl	Bu_3_SnH	**34a:35a**	1:1	97
8	**13**	H	Cl	Bu_3_SnD	**34b:35b**	1:1	82
9	**14**	H	Br	Bu_3_SnH	**34a:35a**	1:1	67 *^e^*
10	**14**	H	Br	Bu_3_SnD	**34b:35b**	1:1	54 *^e^*
11	**18**	H	OTs	Bu_3_SnH	**34a:35a**	1:1	93
12	**18**	H	OTs	Bu_3_SnD	**34b:35b**	1:1	93

*^a^* Reactions were performed on 0.057 mmol (**9** or **18**), 0.094 mmol (**13** or **20**), or 0.078 mmol (**14** or **22**) of substrates (0.029–0.047 M) with 5 equiv. of Bu_3_SnH(D) and 2 equiv. of AIBN in toluene (95 °C, 1–2 h.). *^b^* Determined by ^1^H NMR. *^c^* Isolated yields. *^d^* 1,5-Dideoxy-2-*O*-tosyl-D-ribohexofuranose also formed (19%). *^e^* Hydrodebrominated (2-deoxy) byproducts were also isolated (10–15%).

**Table 2:** Products from 3-*O*-methyl-6-*O*-nitro-1,4-anhydrohexitols *^a^_._*

Entry	Substrate			Reagent	Product	Yield *^b^* (%)
	No	X	Y			
1	**25**	OTs	H	Bu_3_SnH	**37**	63 *^c^*
2	**25**	OTs	H	Bu_3_SnD	**37**	61 *^c^*
3	**32**	Cl	H	Bu_3_SnD	**40**	55
4	**33**	Br	H	Bu_3_SnD	**40**	37 *^d^*
5	**28**	H	Cl	Bu_3_SnD	**40**	63
6	**29**	H	Br	Bu_3_SnD	**40**	47 *^d^*
7	**30**	H	OTs	Bu_3_SnH	**37**	51 *^e^*
8	**30**	H	OTs	Bu_3_SnD	**37**	50 *^e^*

*^a^* Reactions were performed with 0.055 mmol (**25** or **30**), 0.088 mmol (**28** or **32**), or 0.074 (**29** or **33**) of substrates (0.027–0.044 M), 5 equiv. of Bu_3_SnH(D), and 2 equiv. of AIBN in toluene for 1.5 h at 95 °C. *^b^* Isolated yields. *^c^* Compound **38** (22%) also was isolated. *^d^* Hydrodebrominated 2-deoxy byproducts also were isolated (~15–25%). *^e^* Compound **39** (38%) also was isolated.
